# Immunotherapy with Cleavage-Specific 12A12mAb Reduces the Tau Cleavage in Visual Cortex and Improves Visuo-Spatial Recognition Memory in Tg2576 AD Mouse Model

**DOI:** 10.3390/pharmaceutics15020509

**Published:** 2023-02-03

**Authors:** Valentina Latina, Margherita De Introna, Chiara Caligiuri, Alessia Loviglio, Rita Florio, Federico La Regina, Annabella Pignataro, Martine Ammassari-Teule, Pietro Calissano, Giuseppina Amadoro

**Affiliations:** 1 European Brain Research Institute (EBRI), Viale Regina Elena 295, 00161 Rome, Italy; 2 Institute of Translational Pharmacology (IFT), National Research Council (CNR), Via Fosso del Cavaliere 100, 00133 Rome, Italy; 3 IRCCS Santa Lucia Foundation (FSL), Centro di Ricerca Europeo sul Cervello (CERC), Via Fosso del Fiorano 64-65, 00143 Rome, Italy; 4 Institute of Biochemistry and Cell Biology (IBBC), National Research Council (CNR), Via Ercole Ramarini 32, 00015 Rome, Italy

**Keywords:** immunotherapy, Alzheimer’s disease, tau protein, primary visual cortex, vision

## Abstract

Tau-targeted immunotherapy is a promising approach for treatment of Alzheimer’s disease (AD). Beyond cognitive decline, AD features visual deficits consistent with the manifestation of Amyloid β-protein (Aβ) plaques and neurofibrillary tangles (NFT) in the eyes and higher visual centers, both in animal models and affected subjects. We reported that 12A12—a monoclonal cleavage-specific antibody (mAb) which in vivo neutralizes the neurotoxic, N-terminal 20–22 kDa tau fragment(s)–significantly reduces the retinal accumulation in Tg(HuAPP695Swe)2576 mice of both tau and APP/Aβ pathologies correlated with local inflammation and synaptic deterioration. Here, we report the occurrence of N-terminal tau cleavage in the primary visual cortex (V1 area) and the beneficial effect of 12A12mAb treatment on phenotype-associated visuo-spatial deficits in this AD animal model. We found out that non-invasive administration of 12 A12mAb markedly reduced the pathological accumulation of both truncated tau and Aβ in the V1 area, correlated to significant improvement in visual recognition memory performance along with local increase in two direct readouts of cortical synaptic plasticity, including the dendritic spine density and the expression level of activity-regulated cytoskeleton protein Arc/Arg3.1. Translation of these findings to clinical therapeutic interventions could offer an innovative tau-directed opportunity to delay or halt the visual impairments occurring during AD progression

## 1. Introduction

Tau-based immunotherapy programs are underway in clinical trials on human beings for the treatment of Alzheimer’s disease (AD). Multiple lines of evidence have shown that, in addition to the episodic memory and neuropsychiatric manifestations involving the hippocampal CA1 and temporal lobe neocortex, AD is a more globalized disorder encompassing sensory impairments in olfaction, hearing and especially vision [1]. In particular, deficits in visual system function have been described in experimental AD animal models [2,3,4,5,6,7,8,9,10,11,12] and in affected patients [13,14,15,16,17,18,19,20,21,22] consistent with the extensive neuropathology of Aβ plaques and tau neurofibrillary tangles (NFT) in their visual primary and association cortices and sensory system, including the optic nerves and retina themselves. More importantly, in both human beings and in vivo model systems, visuo-spatial complaints and retinal functional deficits are reported to manifest in concomitance [23,24,25,26] or, sometimes, even precede [27,28,29,30,31,32], the occurrence of the signs of memory/learning deterioration traditionally associated with the clinical symptomatology of AD. In this regard, it is widely acknowledged that the functional and anatomical connection of the retina-visual cortex-hippocampus network subserving the integration of higher visual processing into global cognitive performance [33] provides the strong physiological rationale for the direct involvement of visual processing pathways during the slowly progressive clinical course of dementia in AD development [18,34,35]. From the primary visual cortex (V1 area)—which is the main distributor of almost all visual information that reaches other cortical areas [33] in both rodents and primates [36]—visual cues are encoded and processed along both dorsal and ventral visual pathways forming the visual information-processing network which, in turn, is connected to the hippocampus via a multi-synaptic pathway(s) including the secondary visual cortex, temporal cortex, perirhinal/postrhinal cortex and entorhinal cortex [37,38,39,40]. As such, visual neurons participate in the formation of hippocampal-dependent reminiscence, being sensory-driven cortical V1 activity involved in storing the visual component of long-term spatial and episodic memories [41].

We have reported that the neurosensory retina and hippocampus from 6-month-old Tg2576, a well-established preclinical AD animal model which only expresses the human amyloid precursor protein (APP)695 with Swedish mutation (K670N-M671L), respond in parallel to in vivo selective neutralization of 20–22 kDa toxic N-terminal tau fragments (i.e., NH_2_htau) following intravenous (i.v.) injection of the cleavage-specific 12A12 conformational monoclonal antibody (mAb) [12,42]. In this framework, in the present study we explored whether: (i) the neurotoxic accumulation of NH_2_htau also occurred in higher-order cortical visual area(s), in particular in V1, from this AD strain, just as we detected in the retina and hippocampus; and (ii) its in vivo clearance, in response to specific antibody-mediated antagonizing action, translated into functional improvement of visual abilities.

To this aim, the impact of 12A12mAb immunization on the visual-dependent behavioral performance of symptomatic Tg2576 AD mice was evaluated in tight correlation with the assessment of two specific, morphological (dendritic spine density including cofilin 1 phosphorylation) and biochemical (experience-dependent expression of the activity-regulated cytoskeleton protein Arc/Arg3.1) readouts of synaptic plasticity in the primary visual cortex.

## 2. Materials and Methods

### 2.1. Animals and Ethical Approval

All animal experiments complied with the ARRIVE guidelines and were carried out in accordance with the ethical guidelines of the European Council Directive (2010/63/EU); experimental approval was obtained from the Italian Ministry of Health (Authorization n. 1038-2020-PR). This study was carried out according to the principles of the 3Rs (Replacement, Reduction and Refinement).

Heterozygous B6;SJL/Tg2576 mice (Tg-AD) (n = 10–12 per group/treatment), expressing the human amyloid precursor protein (APP) with the Swedish mutation KM670/671NL [43], which causes an increase in Aβ production [44], and their wild-type (Wt) littermates (n = 8–10 per group/treatment) were used at 6 months of age in the immunization regimen. This colony carries mutant alleles that cause functional blindness due to retinal degeneration (Pde6brd1), thus we screened for retinal degeneration within mice used in this study [45]. Genotyping was carried out to confirm the presence of human mutant APP DNA sequence by PCR. 

### 2.2. Immunization Scheme 

The N-terminal tau 12A12 monoclonal antibody (26–36 aa) was produced and purified from hybridoma supernatants according to standard procedures, as previously described [12,42]. 

Mice were randomized into: (1) wild-type mice treated with saline vehicle; (2) age-matched Tg2576 mice treated with saline vehicle; (3) age-matched Tg2576 mice treated with 12A12mAb (30 μg/dose). Animals were infused over 14 days with two weekly injections administered on two alternate days to the lateral vein of the tail. The dose and route of immunization were based on previously published studies by our and other independent research groups using Tg2576 as AD transgenic mouse model [12,42,46]. 

Notably, this immunization regimen was previously demonstrated to successfully deliver in vivo a sufficient amount of biologically active (antigen-competent) anti-tau antibody to promote the clearance of the deleterious NH_2_htau peptide accumulating in animals’ hippocampus and retina and to significantly alleviate their neuropathological signs [12,42]. 

### 2.3. Tissue Collection, Harvesting and Preparation 

For biochemical analysis: 

Two days after the last injection of 12A12mAb [12,42], animals from three experimental groups (wild-type, vehicle-treated Tg-AD, Tg-AD+mAb) were sacrificed by cervical dislocation, brains were collected, primary visual cortices (V1) of both hemispheres [47] were dissected under a dissecting microscope using known landmarks as a guide [48], immediately frozen on dry-ice and, then, stored at −80 °C until use. 

For analysis of experience-dependent markers of V1, animals were sacrificed 1 h after completion of all the tasks included in the visually driven cognitive performance, as reported [49,50]. 

Total protein extracts were carried out as previously reported [12]. Frozen primary visual cortices (V1) were diced and homogenized in ice-cold RIPA buffer (50 mM Tris-HCl pH 8, 150 mM NaCl, 1% NP40, 0.1% SDS, 0.5% sodium deoxycholate) plus proteases inhibitor cocktail (Sigma-Aldrich St. Louis, MO, USA, P8340) and phosphatase inhibitor cocktail (Sigma-Aldrich, P5726/P2850) for 30 min and centrifuged at 4 °C for 20 min at 13,000 rpm. The protein amount was determined by Bradford assay (Protein Assay Dye Reagent Concentrate, Bio-Rad, Hercules, CA, USA).

Crude synaptosomal preparations were obtained from mice of three experimental groups (wild-type, vehicle-treated Tg-AD, Tg-AD+mAb), as reported [51]. Frozen primary visual cortices (V1) were homogenized in 2 mL of homogenization buffer (320 mM sucrose, 4 mM Hepes pH 7.4, 1 mM EGTA, 0.4 mM PMSF, 10 mM NaF, 0.02 M β-glicerophosphate, 1 mM NaVO_3_) plus proteases inhibitor cocktail (Sigma-Aldrich, P8340) and phosphatase inhibitor cocktail (Sigma-Aldrich, P5726/P2850) with 50 strokes in a right-fitting glass dounce tissue grinder. Then the homogenate was centrifuged at 4 °C for 10 min at 1000× *g*, the supernatant was collected and centrifuged at 4 °C for 15 min at 12,000× *g*. The second pellet was resuspended in 1 mL of homogenization buffer and centrifuged at 4 °C for 15 min at 13,000× *g*. Finally, the pellet containing the crude synaptosomal fraction was re-resuspended in 200μL of homogenization buffer.

For morphological analysis: 

Two days after the last injection of 12A12mAb [12,42], sacrificed animals were intracardially perfused with ice-cold phosphate-buffered saline (PBS) 0.1 M, pH 7.4 and, then, with 4% paraformaldehyde (PFA) solution in PBS. After that, brains were carefully removed from the skull, post-fixed in 4% PFA solution in PBS overnight at 4 °C and, then, passed into 30% sucrose solution in PBS for 48–72 h until equilibration. The brains were frozen by immersion in ice-cold isopentane for 3 min before being sealed into vials and stored at −80 °C until use. For analysis of experience-dependent markers of V1, animals were sacrificed 1 h after completion of all the tasks included in the visually driven cognitive performance, as reported [49,50]. 

### 2.4. Western Blot Analysis and Semi-Quantitative Densitometry

Equal amounts of protein extracts (80–150 μg) were loaded for each blot and in each lane regardless of the antibody used and size-fractionated by SDS-PAGE Bis-Tris gel 4–12% (Bolt, Invitrogen). Area-specific samples from different sets of experiments were performed under identical conditions and the average levels of all proteins being studied were roughly comparable between the lysates. After electroblotting onto a 0.2 μm nitrocellulose membrane (Hybond-C Amersham Biosciences, Piscataway, NJ, USA), the filters were blocked in PBS-T containing 5% non-fat dried milk for 1 h at room temperature and, then, incubated with appropriate primary antisera/antibodies diluted in PBS overnight at 4 °C. After 1 h incubation with secondary anti-mouse or anti-rabbit anti-IgG immunoglobulins conjugated with horseradish peroxidase, the blots were developed by using the enhanced chemiluminescence western blotting immunodetection system (ECL) (Thermo Fisher Scientific Waltham, MA, USA West Pico Plus, USA; Amersham Prime, Arlington Heights, IL, USA). The signal detection was performed by using the iBright Imaging Systems (Thermo Fisher Scientific). For statistical analysis, normalization was carried out by using β-actin as internal control of protein loading. Regarding phosphorylated proteins, a signal was reported on corresponding total protein signal. Final figures were assembled by using Adobe Illustrator 10 and Adobe Photoshop 6 and quantitative analysis of acquired images was performed by using Image J 1.4 (http://imagej.nih.gov/ij/ accessed on 18 April 2008). For quantification, we measured the band intensity by using a signal in the linear range.

SDS-PAGE was carried out on 10–20% Tricine gels (Novex, Invitrogen) and electroblotted for 1 h onto 0.1 μm nitrocellulose membrane for the detection of 4 kDa Aβ monomer(s) and its products, as previously described [52]. 

The following antibodies were used:

Tau antibody (BT2) mouse MN1010 ThermoFisher Scientific; Caspase-cleaved protein (CCP) NH_2_-tau antibody rabbit (D25-(QGGYTMHQDQ) epitope, phosphorylation-independent state) [53,54,55]; Arc antibody (C-7) mouse sc-17839 Santa Cruz; anti-SNAP25 antibody (clone SMI 81) mouse 836301 BioLegend; Phospho-p44/42 MAPK (Erk1/2) (Thr202/Tyr204) antibody rabbit 9101 Cell Signaling; p44/42 MAPK (Erk1/2) antibody rabbit 9102 Cell Signaling; phospho-cofilin antibody (Ser3) rabbit 3311 Cell Signaling; cofilin antibody rabbit 3312 Cell Signaling; anti-Alzheimer precursor protein 22C11 (66–81 aa of N-terminus) mouse APP-MAB348 Chemicon; anti-Aβ/APP protein 6E10 (4–9 aa) mouse MAB1560 Chemicon; anti-Aβ amyloid specific (D54D2) rabbit 8243 Cell Signaling; anti-β-actin antibody mouse S3062 Sigma-Aldrich; anti-mouse IgG (whole molecule)-Peroxidase antibody A4416 Sigma-Aldrich (St. Louis, MO, USA); anti-rabbit IgG (whole molecule)-Peroxidase antibody A9169 Sigma-Aldrich (St. Louis, MO, USA). 

### 2.5. Golgi–Cox Staining and Dendritic Spine Analysis 

Two days after the last i.v. injection, mice were sacrificed by deep intraperitoneal (i.p.) anaesthesia and and perfused transcardially with 0.9% saline solution. Brains were collected and immediately immersed in a Golgi–Cox solution (1% K_2_Cr_2_O_7_, 1% HgCl_2_, 0.8% K_2_CrO_4_) at room temperature for 6 days, as previously described [56,57]. On the seventh day, brain tissues were transferred in a 30% sucrose solution for cryoprotection and then sectioned with a vibratome. Coronal sections (100 μm) which contain the primary visual cortex (Bregma −2.5 to −3.2 mm, Interaural 1.3 to 0.6 mm) [48,58] were collected and stained according to the method described by [59]. 

For quantitative analysis [60], a total of 28–30 neurons from eight animals of each experimental group (wild-type; Tg2576; Tg2576+mAb) were used. Layer II/III pyramidal neurons were identified by their distance from pia and their distinct morphologies. Secondary and tertiary dendrites of these neurons were selected for analysis. Dendritic spines were counted on randomly selected 20–30 μm dendritic segments from secondary and tertiary branch order of V1 dendrites using the neuron tracing system (Neurolucida; MBF Bioscience, Williston, VT). Neurons were chosen for analysis based on the following criteria: (1) the quality of Golgi impregnation; (2) the relative isolation of impregnated neurons compared with neighboring impregnated cells; and (3) the position of neurons within the primary visual cortex (V1). Statistical comparisons were made on the groups’ values obtained by averaging the number of spines counted on the neurons of each mouse. Analysis was performed blindly, with the analyzer unaware of the experimental conditions. Spine density was calculated by quantifying the number of spines per measured length of dendrite and expressed as the number of spines per μm (n spines/μm). 

### 2.6. Visual Acuity Test (VAT)

The visual acuity test (VAT) was performed as previously described by Prusky et al. with some modifications [61,62,63,64]. The VAT consisted of three phases carried out in three consecutive days: training day one, training day two and test day. For all the duration of the VAT, mice were subjected to fasting during the dark hours of the light/dark cycle (14 h of fasting before the start of every experimental day).

VA Training phases (day one and two): all mice were handled for 10 min before the beginning of the training phase. Each training session was preceded by a habituation session in which mice were left to freely explore the Y-maze apparatus (long arm: 41.5 × 11.4 × 20 cm; short arms: 34 × 11.4 × 20 cm) for 5 min. Then, on the front wall of the two short arms, two rectangular cards (8 × 30 cm)—a gray colored card (P0) and a black and white striped card with a standard width of 2 cm (P1)—were fixed and presented combined with an associative or a neutral stimulus: the P0 card was associated with a small empty bowl, while the P1 card was associated with a small bowl full of cookie crumbs. During every training session, consisting of 30 trials (30 sec/trial, 10 sec inter-trial interval), mice were placed in the long arm and left free to explore and choose between the two short arms where the P0 (gray) and P1 (2 cm standard striped cards) were randomly located—on the left or right side, respectively—and presented in association with their corresponding stimuli. After completion of the training session, mice were returned to the home cage. 

Training phases were videorecorded with ANY-Maze Software. Scores based on the number of correct answers (consisting in the exploration of P1 card) were manually attributed by a blinded investigator in accordance with the following “criterion”:

- 10 points = *direct* correct answer (mouse went directly to P1);
- 7.5 points = *in between* correct answer (borderline behaviour: mouse went to the middle of the P1-arm, but did not run the whole arm);
- 5 points = *indirect* correct answer (mouse went to P0, right after on P1);
- 0 points = *wrong* arm (mouse did not explore P1).

Only if the animal achieved 70% correct answers (consisting in the exploration of the P1 card) in the training sessions, would it be tested in the next phase.

VA Testing phase: on the third day, mice were subjected to the testing phase, globally consisting of 40 trials. On every consecutive trial, the mouse was released in the long arm and left free to explore and choose between the left or right short arms where a gray colored card (P0) or 4 different black and white striped cards with an increasingly difficult pattern to be discriminated (P1–P4) were randomly placed. 

Four patterns were used (P1–P4) in the 40 trials (10 trials × 4 pairs of cards/session, 30 sec/trial, 10 sec inter-trial interval):

- gray pattern (P0) vs. 2 cm standard striped pattern (P1) card in trials 1–10;
- gray pattern (P0) vs. 1.5 cm striped pattern (P2) card in trials 11–20;
- gray pattern (P0) vs. 1 cm striped pattern (P3) card in trials 21–30;
- gray pattern (P0) vs. 0.5 cm striped pattern (P4) card in trials 31–40.

The test phase was videorecorded with ANY-Maze Software. Test scores based on the number of correct answers consisting in the spontaneous (in the absence of reward) choice of *direct* exploration of P1-P4 cards) were manually attributed by a blinded investigator in accordance with the following criterion:

- 0 point = 0–1 correct answer;
- 2.5 points = 2–3 correct answers;
- 5 points = 4–5 correct answers;
- 7.5 points = 6–8 correct answers;
- 10 points = 9–10 correct answers.

The acuity measurement was assessed on the third experimental day in which the 2 cm black and white wide-striped card was replaced with a narrow-striped one. To sum up, the couples of cards used were progressively more difficult to discriminate: 2 cm striped card vs. gray card, 1.5 cm striped card vs. gray card, 1 cm striped card vs. gray card, 0.5 cm striped card vs. gray card. Parameters analyzed included the number of correct responses and the latency to find the correct cart [62,63]. Visual acuity was finally converted in cycles/degree (c/d) as previously described in the literature [62,63,65]. By considering the distance from the maze divider (34 cm)—the choice point—the maximal visual acuity in wild-type control mice was about 0.4 c/d in line with previous studies [61,64].

### 2.7. Immunofluorescence on Visual Cortex Cryosections

Immunofluorescence on V1 sections was performed according to [66]. For the localization of the primary visual cortex, we relied on coordinates provided by [67,68,69]. In coronal sections, the primary visual cortex starts rostrally at the vertical part of the fimbria hippocampi and reaches as far caudally as the posterior end of the telencephalic hemisphere. In all mice analyzed by immunofluorescence, the complete primary visual cortex of both hemispheres was investigated. For immunostaining, tissue slices were processed and, then, probed overnight at 4 °C with primary antibodies (caspase-cleaved protein (CCP, NH_2_-tau antiserum (D_25_-(QGGYTMHQDQ) epitope, phosphorylation-independent state, 1:100; Arc/Arg3.1, 1:75) in PBS buffer containing 2.5% BSA, 5% NGS and 0.3% Triton X-100, as previously reported [12,42]. Images are representative of at least three independent experiments and were acquired with a spinning disk system for fast fluorescence confocal microscopy, with LED or laser light source from CrestOptics (Crisel Instruments, Rome, Italy). Olympus Confocal Microscope quantitative image analysis was performed by using ImageJ 1.4 (http://imagej.nih.gov/ij/ accessed on 18 April 2008). The relative levels of the molecule of interest were quantified by measuring mean fluorescent intensity (MFI) across a region of interest (ROI), according to [70]. The absence of signal was observed when primary antibodies were omitted.

### 2.8. Histopathological Analysis

For histopathological analysis of the primary visual cortex (V1), animals were intracardially perfused with ice-cold phosphate-buffered saline (PBS) using a 30 mL syringe to remove blood contamination, and the intact brain was isolated, cleaned with PBS with utmost caution not to inflict damage. Brain tissues were dipped in tubes containing 10% formalin solution for the purpose of fixation. Tubes were left at room temperature for 24 h. Later, brain tissues were shifted to melted paraffin wax and solidified. Several tissutal sections of 7 μm thickness were manually trimmed using a microtome (HM325 rotary microtome; Microm, Rijswijk, The Netherland). The tissue slices were subsequently dewaxed, followed by dehydration with increased gradient concentrations of an aqueous alcohol solution. The slices were stained with Gill’s hematoxylin n.2 and eosin dye according to standard protocol, placed on glass slides and observed under a light microscope. In Gill’s Hematoxylin (Bio-Optica CND W01030708) the active chemical specie is the complex formed by hematein (hematoxylin oxidized by potassium iodate) with potassium aluminum sulfate. This complex has a positive charge and is therefore able to bind to anionic sites present in the chromatin histone proteins. Purple nuclei and pink-red cytoplasms are visualized and identified. 

### 2.9. Data Management and Statistical Analysis 

For biochemical and immunofluorescent data analyses, values were expressed as means ± standard error of the mean (S.E.M.) and normalized to wild-type experimental group, considered =1. Statistically significant differences were calculated by one-way analysis of variance (ANOVA) followed by Bonferroni’s post hoc test for multiple comparisons among more than two groups. *p* < 0.05 was accepted as statistically significant (* *p* < 0.05; ** *p* < 0.01; *** *p* < 0.0005; **** *p* < 0.0001). For behavioral experiments, data were analyzed by one-way analysis of variance (ANOVA) followed by Fisher least significant difference (LSD) post hoc test or repeated measures ANOVA. 

Sample size was estimated on the basis of our previously published experiments [12,42] reporting changes in Tg2576 and age-matched wild-type mice after 12A12mAb immunization. An “a priori” estimation to compute the required sample size by a given α power and effect size was carried out by G*Power statistical power analysis (version 3.1.9.4). All statistical analyses were performed using GraphPad Prism 8 software (version 8.4.2).

The theoretical maximal value of the visual acuity in cycles per degree (c/d) was calculated by using the following trigonometric formula [65]: Visual Acuity = ½ ∗ tan^−1^ ∗ W/2 ∗ D where W refers to the width of one cycle (i.e., spacing width of the stripes of 2 cm), and D is the assumed viewing distance from the maze divider (i.e., the choice point), both expressed in cm. 

## 3. Results

### 3.1. Abnormal N-Terminal Cleavage of Tau in Primary Visual Cortex (V1 Area) from Tg2576 AD Mice Is Successfully Antagonized by In Vivo Immunization with 12A12mAb

**Figure 1:**
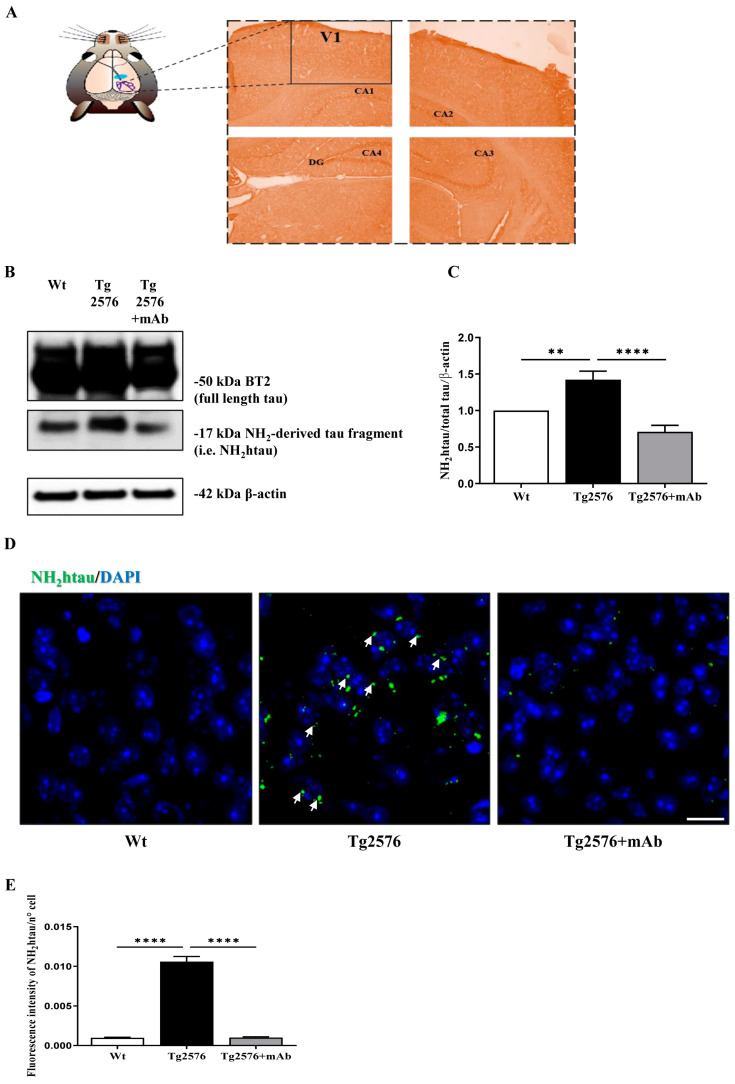
**The neurotoxic 20−22 kDa tau fragment (NH_2_htau) peptide accumulates in the primary visual cortex (V1) of Tg2576 AD mice and it is successfully immunoneutralized following i.v. delivery of 12A12mAb.** (**A**) Representative image of coronal brain slices showing the primary visual cortex (V1, box) of a mouse brain stained with Haematoxylin and Eosin and examined with a light electric microscope at 4×. V1 Scale bar = 50 μm. (**B**) Representative images of SDS-PAGE western blotting analysis (n = 8 animals per each group, 4 males and 4 females for each experimental condition) carried out on protein homogenates of the primary visual cortex (V1) from animals of three experimental groups (littermate wild-type, vehicle-treated Tg2576, Tg-2576+mAb) with BT2, the pan-tau antibody directed against the 194–198 amino acids of full-length protein, to detect the steady-state expression level of the neurotoxic 20–22 kDa NH_2_htau peptide. (**C**) Semi-quantitative densitometry of the intensity signals of bands was carried out following normalization with β-actin level used as loading control. Values are from at least three independent experiments and statistically significant differences were calculated by one-way ANOVA followed by Bonferroni’s post hoc test for multiple comparisons among more than two groups. *p* < 0.05 was accepted as statistically significant. (**D**) Representative images of immunofluorescence analysis (20×) showing the increase in the punctate, dot-like staining (arrows) of the NH_2_htau (green channel) in the primary visual cortex (V1) from Tg2576 AD mice in comparison with age-matched controls and its significant downregulation following 12A12mAb immunization (n = 6 animals per each group, 3 males and 3 females for each experimental condition). Scale bar = 25 μm. (**E**) Fluorescence intensity quantification of the NH_2_htau staining in the V1 area from three experimental groups (sample size: analyzed neurons/animal = 1050, n = 6). Values are from at least three independent experiments and statistically significant differences were calculated by one-way ANOVA followed by Bonferroni’s post hoc test for multiple comparison among more than two groups. *p* < 0.05 was accepted as statistically significant (** *p* < 0.01; **** *p* < 0.0001).

To explore the possible causal link between the chronic elevation of pathogenic truncated tau along the visual pathway and the in vivo disruption of visual–cognitive synaptic plasticity, we first evaluated whether the expression of the neurotoxic, N-terminal 20–22 kDa tau fragment occurring in the retina and humor vitreous of 6-month-old Tg2576 mice [12] may be also detected in their higher centers of vision, in particular in the primary visual cortex (V1). In contrast to the tauopathy animal model that does not show amyloidosis, Tg2576 overexpressing the APP695 isoform with the Swedish mutation APP KM670/671NL (TgHuAPP695swe) displays progressive hippocampus-based synaptic and cognitive impairments, ocular disturbances depending on both Aβ and tau pathologies [12,42,46,51,71] and, thus, better mirrors important aspects of human AD pathology [71]. In this framework, we chose to analyze the V1 area of the left and right hemispheres (Figure 1A) since it: (i) is the main distributor of almost all visual information that reaches other cortical areas [33] with neural circuitry for vision both in rodents and primates [36,72]; (ii) subserves important visual processing functions, such as orientation and spatial frequency selectivity [73,74]; (iii) undergoes alterations in the synaptic plasticity and processing pathways of visual information in the AD mouse model and affected patients [75,76,77]; and (iv) is functionally modulated by tau expression in adult and old mice [78]. To this aim, western blotting SDS-PAGE analyses followed by semi-quantitative densitometry were carried out on crude synaptosomal fractions of V1 by probing with BT2 (194–198 aa), a commercial tau antibody reacting against the N-terminal end of tau. As shown in Figure 1B,C, we found that the endogenous steady-state expression level of the toxic NH_2_htau peptide was significantly increased in V1 samples from 6-month-old Tg2576 AD mice in comparison to their wild-type littermate controls (one-way ANOVA followed by Bonferroni’s post hoc test; ** *p* < 0.01 Tg2576 vs. wild-type) and successfully immunodepleted by non-invasive i.v. administration of 12A12mAb (one-way ANOVA followed by Bonferroni’s post hoc test; **** *p* < 0.0001 Tg2576+mAb vs. Tg2576), in line with our corresponding findings on both the retina and hippocampus from this strain [12,42].

Similar observations were also obtained by immunofluorescence analysis (Figure 1D,E) with the caspase-cleaved protein (CCP)-NH_2_tau antiserum (D_25_-(QGGYTMHQDQ) epitope, phosphorylation-independent state [12,42] showing that the punctate staining was strongly increased in coronal V1 sections from Tg2576 AD mice in comparison to their wild-type controls (one-way ANOVA followed by Bonferroni’s post hoc test; **** *p* < 0.0001 Tg2576 vs. wild-type) and that the intracellulary positive labeling was significantly reduced following 12A12mAb treatment (one-way ANOVA followed by Bonferroni’s post hoc test; **** *p* < 0.0001 Tg2576+mAb vs. Tg2576). 

Taken together, these results show for the first time that the cleavage at the N-terminal tau with the release of diagnostic 20–22 kDa toxic neuropeptide (i.e.,NH_2_htau) is not only restricted to the retina and associated ocular structures of 6-month-old Tg2576 AD mice [12], but is also extended to their primary visual cortex (V1 area), in agreement with studies reporting that the accumulation of other pathogenic misfolded and/or hyperphosphorylated tau species is detectable along the entire visual system both in different preclinical AD animal models and AD subjects [7,79,80,81,82,83]. More importantly, the NH_2_htau fragment is successfully immunodepleted in the V1 area by 12A12mAb administration, which also exerts an anti-amyloidogenic effect by reducing the local expression levels of APP/Aβ (Supplementary Figure S1), just as we reported to occur in the hippocampus and in the retina of this transgenic strain [12,42].

### 3.2. In Vivo Functional Alterations of Visuo-Spatial Skills Are Recovered by 12A12mAb Immunization in Tg2576 AD Mice 

Deficits in visual discrimination, in particular in visual acuity [84,85], associated with the deterioration of pyramidal neurons located in the primary visual cortex [86] have been described in transgenic mouse models of AD and other tauopathies [75,87,88] and in affected patients [14,15]. 

**Figure 2:**
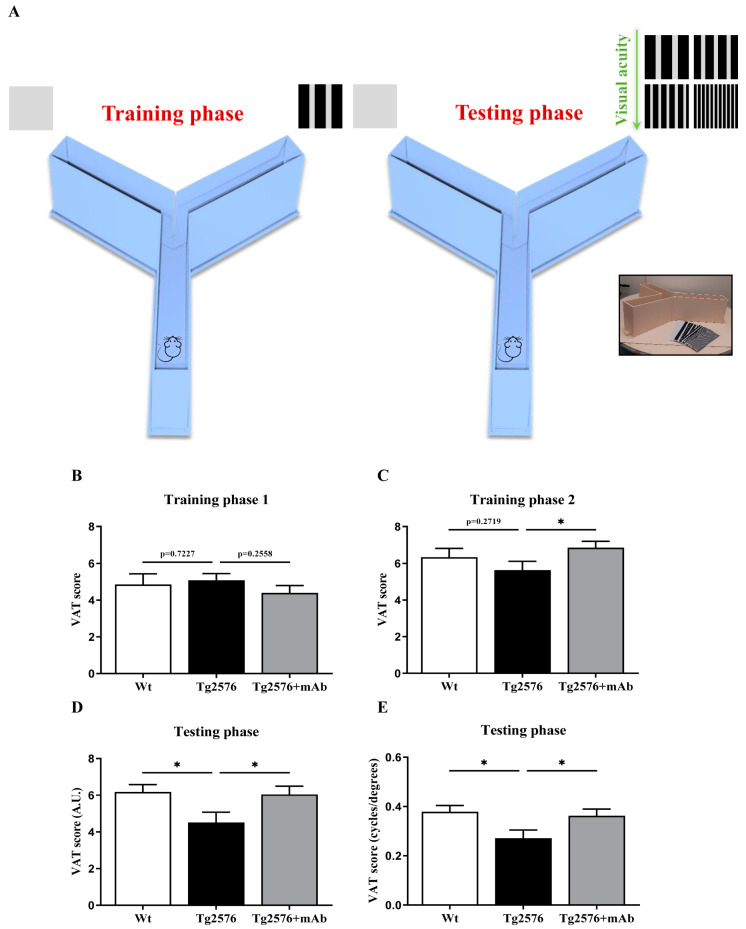
**The low visual acuity of Tg2576 AD mice is significantly improved following 12A12mAb immunization.** (**A**) Schematic components of the visual acuity testing apparatus. (**B**–**E**) Male Animals (n = 33) from three experimental groups (littermate wild-type = 10, vehicle-treated Tg2576 = 11, Tg2576+mAb = 12) were tested for their visual acuity in the Prusky’s test, as described (see Materials and Methods for details). Histograms show the visual acuity test (VAT) training average score in the “training phase” for day 1 (**B**) and for day 2 (**C**) and the visual acuity test average score in “testing phase” (**D**). Individual performance scores were converted in cycles/degree (c/d) according to the standard trigonometric formula [65] on assumed viewing distance (in cm) and results were shown in (**E**). Values are from at least three independent experiments and statistically significant differences were calculated by one-way ANOVA followed by Fisher least significant difference (LSD) post hoc test. *p* < 0.05 was accepted as statistically significant (* *p* < 0.05). A.U., arbitrary units.

Thus, having established that the aberrant expression of truncated tau was elevated not only in the retina but also in the V1 area from Tg2576 AD mice, we further assessed whether its occurrence into the visual pathway was correlated with their poor performance in visual acuity and whether its in vivo immunoneutralization following 12A12mAb treatment was beneficial to this classical vision disturbance associated with AD symptomatology. To achieve this goal, we used an adaptation of the procedure employed by Prusky et al. [61] to train mice in visual detection and pattern discrimination tasks and, then, to test their visual acuity proficiency in a three-phase behavioral paradigm. The visual acuity test (VAT) consists of two phases: the training phase and test phase. In the training phase (two days), mice were trained to associate a visual stimulus (card with standard striped pattern, P1) with a reward. In the test phase, mice were then challenged to discriminate visual stimuli consisting of cards with striped patterns of increasing difficulty levels (P1-P4). Thus, animals from three experimental groups (littermate wild-type, vehicle-treated Tg-AD, Tg-AD+mAb) were asked to discriminate between a “grey card” and a “cue card” that contains a pattern of vertical black and white stripes of different width and is associated with a food reward. The wider cue black card (2 cm wide stripes) was randomly and sequentially changed to gradings of 1.5, 1, 0.5 width (Figure 2A). The performance in visual acuity was evaluated at the release site (point of choice) when the grading of the cue cards was sequentially altered to test animals’ ability in perceiving them as grey or resolved as stripes [62,63,65].

Figure 2B–E summarises the results of experimental tests when the different grading of the cue cards was progressively more narrow. In the training phases (Training 1 and Training 2), repeated measures ANOVA analysis on average scores of data (expressed in arbitrary units, A.U.) from day one and two demonstrated no effect of groups (F_(2, 33)_ = 0.17; *p* = 0.84), but only a significant day effect (F_(1, 33)_ = 25.79; *p* = 0.00001). Consistently, on training day one (Figure 2B, Training 1), one-way ANOVA analysis followed by a Fisher least significant difference (LSD) test for post hoc pair comparisons showed that all three experimental groups had similar visual skills (*p* > 0.05 for all comparisons). On the contrary, on training day two (Figure 2C, Training 2), a treatment effect was detected because the Tg2576+mAb cohort displayed a significant improvement in their visual-dependent associative performance in comparison with their vehicle-treated counterparts (training day two, *p* = 0.045, * *p* < 0.05 Tg2576+mAb vs. Tg2576), whereas no difference was contextually observed between Tg2576 AD mice and their littermate wild-types (*p* > 0.05 Tg2576 vs. wild-type). In the test phase (Figure 2D), one-way ANOVA analysis on average scores of data from P1 to P4 phases showed that visual acuity significantly differed among three groups. More importantly, and consistent with strong accumulation of the neurotoxic NH_2_htau in both the animals’ retina [12] and V1 area (Figure 1), an impaired performance with robust reduction in their visual discrimination was measurable in Tg2576 AD mice, showing a lower average VAT score when compared to age-matched controls (one-way ANOVA post hoc Fisher LSD test; *p* = 0.027, * *p* < 0.05 Tg2576 vs. wild-type). These functional results fit well with the low capacity in visual acuity detected in AD subjects [84,85] along with other in vivo studies showing that several independent lines of transgenic AD mice have poorer visual stimuli-based behaviour in the absence of any change in the odor preference and anxiety [89,90]. Remarkably, following i.v. administration of 12A12mAb, Tg2576 animals exhibited a significant increase in the average VAT score with respect to the not-vaccinated cohort, indicating that immunization rescued their visual acuity defects nearly up to baseline conditions (one-way ANOVA post hoc Fisher LSD test; *p* = 0.029, * *p* < 0.05 Tg+mAb vs. Tg2576).

As predicted [62,63,91,92] discrimination performance of all three experimental groups progressively dropped as the pattern of the black and white cue stripes decreased (width varies from 2 cm to 0.5 cm black). By repeated measures ANOVA analysis of “raw” data test scores on consecutive P1, P2, P3 and P4 phases (Supplementary Figure S2), we also found a significant group effect (F_(2, 33)_ = 3.62; *p* = 0.038). In agreement, a post hoc Fisher LSD test showed that a significant reduction in the VAT score in the P1 phase took place in Tg2576 when compared to littermate wild-type mice (*p* = 0.026, Tg2576 vs. wild-type), and that this diminution was successfully relieved in Tg2576+mAb (*p* = 0.045, Tg2576+mAb vs. Tg2576). Moreover, and more importantly, the same trend emerged in the P2 phase because we detected a sizeable, although not significant, decrease in the VAT score when not-vaccinated Tg2576 mice were compared with their controls (*p* = 0.07, Tg2576 vs. wild-type) with a moderate upregulation following antibody delivery in the Tg2576+mAb group (*p* = 0.06, Tg2576+mAb vs. Tg2576).

The visual acuity was converted in cycles/degree (c/d) score (see Materials and Methods) and results were shown in Figure 2E. Consistently, the untreated wild-type mice showed maximal visual acuity of about 0.378 c/d, in line with previous findings [62,63,64,65], in contrast to the significantly impaired performance (0.271 c/d) of the age-matched Tg2576 cohort (one-way ANOVA followed by post hoc Fisher LSD test; * *p* < 0.05 Tg2576 vs. wild-type). Interestingly, in concomitance with successful immunoneutralization of the pathogenic NH_2_htau in their V1 sensory cortex (Figure 1), antibody immunization of Tg2576 mice significantly ameliorated their visual acuity, which rose up to the average value of 0.362 c/d (one-way ANOVA followed by post hoc Fisher LSD test; * *p* < 0.05 Tg2576+mAb vs. Tg2576). 

Taken together, this study extends previous findings of visuo-spatial alterations in mouse models of AD and other tauopathies [4] by demonstrating uniquely that the accumulation of a specific, pathogenic N-terminal-cleaved form of tau (i.e., NH_2_htau) in the V1 area of Tg2576 AD mice translates into functional impairments of their visual performance (i.e., reduced visual acuity) and that this diminution is significantly recovered by treatment with 12A12mAb. 

### 3.3. Treatment of Tg2576 AD Mice with 12A12mAb Normalizes the Changes in the Neural Expression Pattern of Experience-Dependent Markers Closely Coupled to the Synaptic Plasticity of the V1 Area

**Figure 3:**
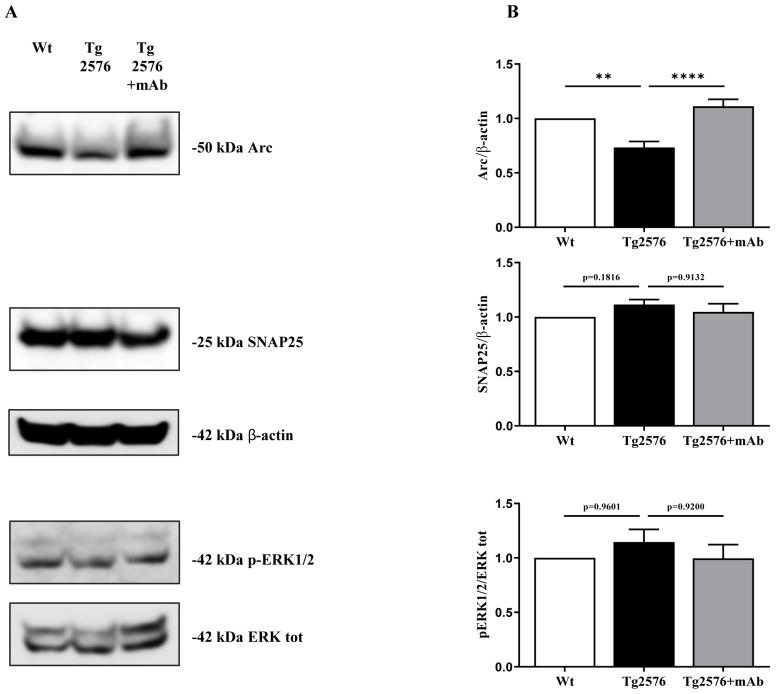
**Effect of 12A12mAb immunization on expression pattern of experience**−**dependent markers of V1 plasticity from Tg2576 AD mice**. (**A**) Representative images of SDS-PAGE western blotting analysis (n = 8 animals per each group, 4 males and 4 females for each experimental condition) carried out on protein homogenates of the primary visual cortex (V1) from animals of three experimental groups (littermate wild-type, vehicle-treated Tg2576, Tg2576+mAb) with antibodies reported alongside the blots. Arrows on the right side indicate the molecular weight (kDa) of bands calculated from migration of standard proteins. (**B**) Semi-quantitative densitometry of the intensity signals of bands was carried out following normalization with β-actin level used as loading control. For the determination of the phosphoERK1/2 level, the phospho-ERK 1/2/total ERK1/2 ratio was calculated. Values are from at least three independent experiments and statistically significant differences were calculated by one-way ANOVA followed by Bonferroni’s post hoc test for multiple comparisons among more than two groups. *p* < 0.05 was accepted as statistically significant (** *p* < 0.01; **** *p* < 0.0001).

To further corroborate our functional results, the in vivo biochemical response of neurons to visual stimulation was verified in 6-month-old animals from three experimental groups (littermate wild-type, vehicle-treated Tg-AD, Tg-AD+mAb) by evaluating the synaptic alterations of the primary visual cortex in the experience-driven expression of the immediate early gene Arc (activity-regulated cytoskeleton protein). Arc (also known as Arg3.1) is an activity-dependent regulator of excitatory synaptic transmission interacting with specific effector proteins in different neuronal compartments [93] and its induction provides a convenient readout of long-term synaptic plasticity into the primary visual cortex (V1), which is functionally connected to the stimulated eye [49,50,94,95,96]. Thus, to track the neuronal responses to visual inducement in animals undergoing behavioral task, we took advantage of the experimental approach combining their environmental challenge with post-mortem assessment of the immediate early gene Arc activation in the V1 area [97]. To this aim, mice tested for their visual abilities were sacrificed 1 h after completion of discrimination trial, synaptic-enriched homogenates were prepared from the V1 area and, then, analyzed by western blotting SDS-PAGE with specific Arc/Arg3.1 antibody, followed by semi-quantitative densitometry. As shown in Figure 3A,B and in line with previous results reporting that Arc/Arg3.1 activation following sensory incentive was greatly impaired in the visual cortex from APP/PS1 AD mice [98,99,100], the synaptic response to physiological consolidation of visual experience was disrupted in 6-month-old Tg2576 AD animals when compared to their littermate wild-types (one-way ANOVA followed by Bonferroni’s post hoc test; ** *p* < 0.01 Tg2576 vs. wild-type). Interestingly, a robust increase in Arc/Arg3.1 expression was detectable following 12A12mAb immunization (one-way ANOVA followed by Bonferroni’s post hoc test; **** *p* < 0.0001 Tg2576+mAb vs. Tg2576), indicating that immunization of the Tg2576 AD cohort markedly recovered the normal sensory-dependent functional plasticity of their visual cortices. On the contrary, no statistically significant differences (*p* > 0.05 among three experimental groups) were found by probing protein extracts with antibodies for phospho-extracellular signal-regulated kinase 1/2 (pERK1/2) and synaptosomal-associated protein 25 kDa (SNAP-25), two other activity-dependent regulators known to be crucially involved in visual cortex plasticity [78,101,102]. 

**Figure 4:**
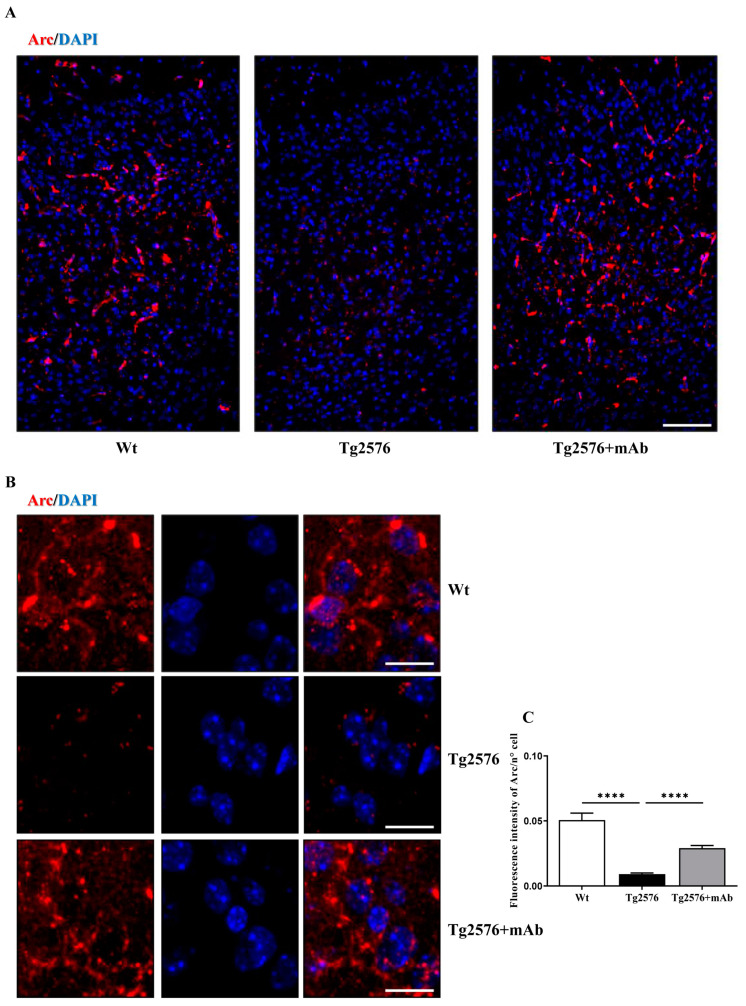
**Impaired Arc/Arg3.1 protein expression in the V1 area in response to visual stimulation of Tg2576 AD mice is rescued by 12A12mAb administration**. (**A**,**B**) Immunohistochemical staining showing Arc/Arg3.1 (red channel) and DAPI nuclear stain (blue channel) in the primary visual cortex (V1) from animals (n =6 animals per each group, 3 males and 3 females for each experimental condition) of three experimental groups (littermate wild-type, vehicle-treated Tg2576, Tg2576+mAb). Original magnifications: (**A**), 20×; (**B**), 40×; Scale bar = 50 μm (20×); 25 μm (40×). (**C**) Fluorescence intensity quantification of the Arc/Arg3.1 positivity in the V1 area from three experimental groups (sample size: analyzed neurons/animal = 1050, n = 6). Values are from at least three independent experiments and statistically significant differences were calculated by one-way ANOVA followed by Bonferroni’s post hoc test for multiple comparisons among more than two groups. *p* < 0.05 was accepted as statistically significant (**** *p* < 0.0001).

To confirm that antibody-mediated neutralization of the neurotoxic NH_2_htau could positively influence the expression level of Arc/Arg3.1 in individual neurons of the V1 population, we also performed immunohistochemical analysis on coronal brain sections from the three experimental groups that were sacrificed 1 h after the end of visual task. As shown (Figure 4A–C), the cytosolic extent of Arc/Arg3.1 dot-like labeling was strongly lower in 6-month-old Tg2576 AD mice when compared to their age-matched controls (one-way ANOVA followed by Bonferroni’s post hoc test; **** *p* < 0.0001 Tg2576 vs. wild-type). These findings are in agreement with previous studies reporting that only a sizeable proportion of Arc-positive neurons was responsive to behavioral stimulus in brains from other tauopathy animal models [98,103,104]. This diminution in activable neurons in the V1 circuits was more likely to contribute to the loss of functional response to visual stimulation [94,105,106] as we recorded when AD animals were contextually challenged in a vision-based behavioral paradigm. On the contrary, the amplitude of Arc responses was largely upregulated following 12A12mAb delivery (one-way ANOVA followed by Bonferroni’s post hoc test; **** *p* < 0.0001 Tg2576+mAb vs. Tg2576), indicating that the percentage of neurons activated by visual incentive in relevant cortical networks in the Tg2576 AD cohort was significantly higher than in their not-vaccinated counterparts, consistent with their improved performance following sensory experience (Figure 2). 

To rule out the possibility that this functional loss could be ascribed to unspecific neuronal death, V1 sections were also stained with neuron-specific nuclear antigen (NeuN), a neuron-specific marker localized in the cell nucleus and cytoplasm of postmitotic neurons [107]. Interestingly (Supplementary Figure S3), no significant reduction in NeuN-positive cells was found in Tg2576 mice, even though a sizeable but not significant reduction in area (indirect index of cell volume) was detectable in comparison with their non-transgenic controls. These results confirmed that a strong reduction in neuronal activity-induced gene expression rather than a general neuronal loss took place in the V1 area of this AD animal model at 6 months of age, further supporting the notion that in this neuronal population, pathogenic NH_2_htau fragments critically affected the level expression of Arc in response to visual stimulation. No change in neuronal density was also detected following 12A12mAb treatment. 

Collectively, our data indicate that: (i) the performance of visual discrimination and the ensuing experience-dependent stimulation of the visual cortex are significantly impaired in 6-month-old Tg2576; and (ii) the loss of visually evoked stimulation due to, at least in part, the accumulation of the pathogenetic NH_2_htau along their neurosensorial network including the V1 area, occurs in the absence of frank neuronal loss and positively responds to treatment with 12A12mAb. These results complement and extend recent findings indicating that tau is able to modulate the visual plasticity in vivo [78] and that their behavioral correlates are disrupted in V1 synaptic circuits even in the early stage of tauopathy in animal model [88].

### 3.4. 12A12mAb Treatment Attenuates the Changes in Dendritic Spine Density and Cofilin Phosphorylation Occurring in the Primary Visual Cortex of Tg2576 AD Mice

The visually driven plasticity in cortical circuits and the dynamics of dendritic spines are mutually dependent since the functional alterations in visual experiences are strictly linked with structural changes of synaptic connectivity in the mouse primary visual cortex [108,109,110,111]. Furthermore, the phosphorylation status of the actin-binding protein cofilin 1, a major regulator of actin dynamics in dendritic spines modifications involved in AD synaptotoxicity [112], subserves the modulation in plasticity of the mouse primary visual cortex, both in development and in adult life [113,114,115].

**Figure 5:**
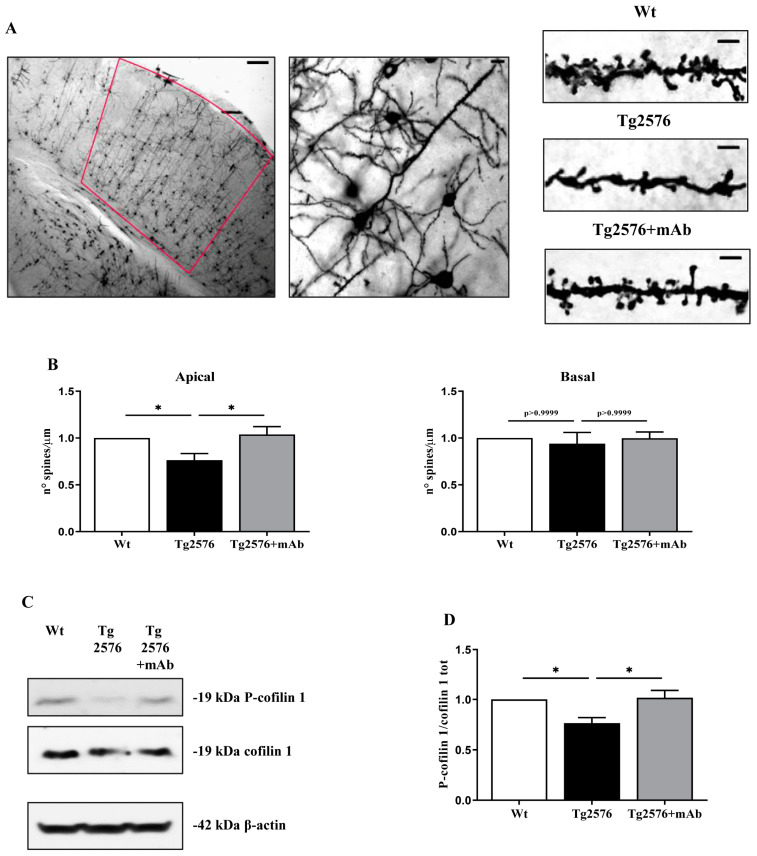
**Treatment with 12A12mAb is protective against the dendritic spine density loss occurring in the primary visual cortex (V1) from Tg2576 AD mice in concomitance with deficits in their vision.** (**A**) Representative micrographs of a coronal hemisection at the level of the primary visual cortex (V1) impregnated using the Golgi–Cox method showing dendritic segments from animals (n = 6 animals per each group, 3 males and 3 females for each experimental condition) of three experimental groups (littermate wild-type, vehicle-treated Tg2576, Tg2576+mAb). Original magnifications of different focus were merged using the CombineZP software (version 1.0) to obtain the representative images (from left to right): 5×, 20×, 100×; Scale bar = 250 μm (5×); 25 μm (20×); 5 μm (100×). (**B**) Histograms depict the morphometric analysis of the dendritic spine density from three experimental groups. Values are expressed as number of spines per 1 μm segment (n spines/μm). Statistically significant differences (comparisons were made on single mouse values obtained by averaging the number of spines counted on neurons of the same mouse) were calculated by one-way ANOVA followed by Bonferroni’s post hoc test for multiple comparisons among more than two groups. *p* < 0.05 was accepted as statistically significant. (**C**) Primary visual cortex (V1) homogenates from animals (n = 6 animals per each group, 3 males and 3 females for each experimental condition) of three experimental groups (littermate wild-type, vehicle-treated Tg2576, Tg2576+mAb) were analyzed by SDS-PAGE western blotting with antibodies reported alongside the blots. Arrows on the right side indicate the molecular weight (kDa) of bands calculated from migration of standard proteins. β-actin was used as loading control. (**D**) Semi-quantitative densitometry of the intensity signals of bands was carried out following normalization of phospho-cofilin/total cofilin ratio. Values are from at least three independent experiments and statistically significant differences were calculated by one-way ANOVA followed by Bonferroni’s post hoc test for multiple comparisons among more than two groups. *p* < 0.05 was accepted as statistically significant (* *p* < 0.05).

Thus, to evaluate whether the impaired behavioral response of 6-month-old Tg2576 AD mice to visual experience had also structural correlates at the synaptic level in their primary visual cortex and whether these alterations were both positively modulated following 12A12mAb administration, Golgi–Cox impregnation was carried out on all the pyramidal V1 neurons in order to stain and analyze the dendritic spine arborization [116]. Figure 5A,B shows representative micrographs and relative quantification of the dendritic spine density (number of spines per unit length) along both apical and basal compartments of individual V1 pyramidal neurons from animals of three experimental groups (littermate wild-type, vehicle-treated Tg-AD, Tg-AD+mAb). As expected, a prominent spine loss was detectable at the age of 6-months in apical compartments of V1 neurons from Tg2576 when compared to age-matched controls (one-way ANOVA followed by Bonferroni’s post hoc test; * *p* < 0.05 Tg2576 vs. wild-type). Importantly, in the 12A12mAb-immunized AD group, the apical spine density was significantly ameliorated, nearly up to the baseline level (one-way ANOVA followed by Bonferroni’s post hoc test; * *p* < 0.05 Tg2576+mAb vs. Tg2576), indicating that treatment was strongly effective in blocking/preventing the dendritic degeneration in the V1 area. Interestingly, no statistically significant difference (*p* > 0.05) was detected among three experimental cohorts when spines were counted in their basal compartment, in line with similar results by our and other research groups on CA1 pyramidal neurons from this genetic background [42,51,117,118]. 

Next, we evaluated whether the morphological alterations of spine density detected in the primary visual cortex of 6-month-old animals from three experimental groups (littermate wild-type, vehicle-treated Tg-AD, Tg-AD+mAb) were also associated with corresponding modulation in its biochemical correlate, such as the expression level and phosphorylation pattern of cofilin 1 [113,114,115]. In this regard, western blotting SDS-PAGE analyses followed by semi-quantitative densitometry were carried out on whole V1 homogenates by probing with specific antibody for position 3 (Ser-3-phosphorylated) of cofilin 1 (P-cofilin), a residue known to critically regulate its activity [112]. As shown in Figure 5C,D, the P-cofilin 1 signal significantly decreased in 6-month-old Tg2576 AD animals when compared with littermate wild-types (* *p* < 0.05 Tg2576 vs. wild-type), in line with previous results in AD mouse models, such as APP/PS1 and 3 × Tg and affected patients [119,120,121,122,123,124]. Interestingly, the immunoreactivity intensity of P-cofilin 1 marker was strongly upregulated in the Tg2576 AD cohort following 12A12mAb immunization (* *p* < 0.05 Tg2576+mAb vs. Tg2576), indicating that treatment with the antibody exerted a protective effect on V1 dendritic actin dynamics. No significant change in the amount of total cofilin 1 was detected among three animal cohorts (*p* > 0.05).

Overall, these results indicate that the targeting/removal of the pathogenetic NH_2_htau following 12A12mAb delivery improves the visuo-spatial skills in Tg2576 AD mice in association with synaptic alterations in dendritic spine density and plasticity in their V1 cortical areas.

## 4. Discussion

The main findings of the present study are the following: (i) the chronic accumulation of the neurotoxic NH_2_htau fragment along the neurosensorial network (eye–brain) of 6-month-old Tg2576 AD mice disrupts their visual performance along with key readouts of synaptic plasticity in the vision-related cortical V1 area; (ii) tau-directed immunotherapy following non-invasive i.v. delivery of 12A12mAb significantly improves in vivo the phenotype-associated deficits in visuo-spatial skills, as demonstrated by means of different but complementary experimental approaches, including behavioral (visual acuity test), biochemical (induction of experience-dependent expression of Arc) and morphological (dendritic spine density) evaluations.

Compelling clinical and neuropathological evidence has demonstrated that a global deficit in acquisition and integration of visual information crucially contributes to impairment of episodic memory in AD [18,124,125,126,127,128,129,130] and mild cognitive impairment (MCI) subjects [131,132], in association with a high burden of tau and β-amyloid in their visual system [83,84,85,133,134,135]. A positive correlation between the disruption of connectivity in the visual functional circuitry and the overall amnestic severity has also been recently confirmed in patients suffering the AD spectrum [136,137,138]. Additionally, a poor visual performance, in particular in visual acuity, has been detected in AD subjects [84,85] and in different animal models in concomitance with the progressive development of the characteristic disease-associated histological hallmarks into their visual system [8,11,89,90,139,140]. In this framework, by taking advantage of a well-established protocol of a discrimination test based on visual cues [61,62,63,64,91,92,141,142,143,144,145,146] that involve the visual cortex and possibly the V1 area, we report that: (i) 6-month-old Tg2576 AD mice showing a high burden of truncated tau in the V1 area are impaired in discriminating between two different spatial frequencies when challenged in a visual acuity task; and (ii) in vivo treatment with antagonizing 12A12mAb significantly improves their visuo-spatial skills, since immunized animals display a better performance in making a decision between two visual stimuli and, then, in reaching the incentive cue faster than not-vaccinated counterparts. Importantly, the visuo-spatial skills of the experimental mouse strain we employed are not influenced by their genetic background, although the B6;SJL/Tg2576 strain [43] *per se* carries the Pde6brd1 mutation (RD1), causing retinal degeneration and progressive blindness. Selected mice used to establish our colony were confirmed to be free of the RD1 allele contamination and, then, suitable for spatial memory tests that rely on the use of visual cues [45,147]. Furthermore, the changes in visual plasticity we detected cannot be explained by differences in animals’ behavioral state, since Tg2576 mice turn out to be even more active than the wild-type controls starting from 6 months of age by displaying an increased locomotor activity [148,149,150,151,152] known to enhance stimulus-specific plasticity in the adult visual cortex [153]. Our in vivo findings are in line with previous studies reporting that the deposition of β-amyloid plaques and neurofibrillary tangles—two hallmark lesions of AD brains—are also detected throughout the entire visual system [154,155,156,157,158], in particular in the primary visual cortex [157,158] and in correlation with the loss of V1 pyramidal neurons [158] in affected subjects. Interestingly, compelling neuro-ophthalmic investigations have shown that the retinal or optic nerve deterioration—even though being associated with disturbances of vision [159]—could be *per se* negligible for explaining the prominent visual deficits afflicting affected patients, especially when compared to extensive pathologic changes co-occurring in their visual association cortex [18,76,77,160]. Additionally, subjects with MCI or full-blown frank dementia, exhibit dense neurofibrillary tangles (NFTs), neuropil threads, and tau-immunoreactive neurites surrounding neuritic plaques (NPs) in their primary visual cortex (Brodmann Area 17) [157,161,162] and visual association cortex (Brodmann area 19) [158], indicating that the occurrence of tau pathology in these high-order structures is more likely to be causative for the early alterations of visual signal processing occurring during the AD progression. Finally, our results also fit well with more recent studies indicating that physiopathological tau protein *per se* modulates the plasticity in primary visual cortex in adult and old mice, both in healthy [78] and in disease [88]. In this regard, it is worth stressing that V1 is not the only relevant structure of the visual system, because we cannot rule out that the decrease in the visual acuity performance we found in Tg2576 mice could be ascribed to alterations in more than one structure or upstream pathway of the visual system beyond the V1 area, being the pathological accumulation of NH_2_htau peptide also detectable in their retina and humor vitreous [12]. Moreover, and more importantly, the low (sensory retina) [12] and higher visual centers (primary visual cortex) are both directly targeted in vivo by 12A12mAb administration with great benefit to the vision of the immunized AD cohort.

Another relevant observation of the present work is that immunization of symptomatic Tg2576 mice with 12A12mAb normalizes, even up to the control values, the steady-state expression levels of Arc/Arg3.1 and P-cofilin 1, two known activity-dependent regulators crucially involved in primary visual cortex plasticity [49,50,78,94,95,96,113,114,115]. In this regard, several pieces of conflicting data point toward a potential role of Arc/Arg3.1 in subserving the tau-mediated neurodegeneration. On one hand, no relationship between tau insoluble neurofibrillary tangles (NFT) and differential Arc expression in the cortical V1 area has been reported in rTg4510, a transgenic mouse model of tauopathy overexpressing the P301L mutant form of human tau driven under a tetracycline-operon responsive element that is suppressible with doxycycline (DOX) [97]. On the other hand, an overall diminution in the neuronal amount of the Arc mRNA has been reported to take place at 11–12 months of age in the brain from the same strain, both at baseline and after environmental enrichment, and this level was significantly recovered following the transgene suppression by means of DOX administration [103]. In this context, our findings are more consistent with this latter evidence by demonstrating that the accumulation of unaggregated NH_2_htau fragments significantly disrupts the Arc experience-dependent induction in the V1 area of 6-month-old Tg2576 AD mice in a way that is responsive to 12A12mAb treatment. Overall, our in vivo observations further support the conclusion that: (i) the elevation of Arc/Arg3.1 is connected with functional visual impairments and not a mere consequence of tau pathology; and (ii) the most toxic soluble species, including the truncated ones (i.e., NH_2_htau), but not the neurofibrillary aggregates are *per se* responsible for the general progressive deterioration of the functional network connectivity during the AD development and that their deleterious effects are reversible in vivo.

Recent studies have identified the experience-dependent turnover of dendritic spine by actin-binding protein cofilin 1 as a reliable anatomical readout of mouse primary visual cortex plasticity, both in development and in adult life [113,114,115]. However, the relevance of pathological changes in phosphorylation state of cofilin 1 in driving the AD progression is still controversial [112,163]. Bidirectional shifts in phosphorylated/inactive vs. dephosphorylated/active forms of cofilin 1 in the brain critically contribute to the loss of dendritic spines and synapses underlying the AD-associated cognitive impairment [121,164,165,166]. On one hand, elevated levels of inactive/phosphorylated form of cofilin-1 are detectable in brain specimens from AD mouse models, such as the APP/PS1 model, and AD affected patients [121,167,168,169]. On the other hand, deficits in synaptic plasticity in the AD progression are also linked with the active/dephosphorylated species of cofilin-1 which aggregate into aberrant cofilin-actin rods, leading eventually to an axonal trafficking jam, blockage of intracellular transport of mitochondria, loss of dendritic spines and synapse starvation [170,171,172,173,174]. Interestingly, in several experimental AD animal models including Tg2576 mice, alterations in dendritic spine density and complexity occurring in the CNS are tightly paralleled by concomitant and corresponding changes occurring in the neurosensorial circuit, in particular in the retina, demonstrating that the eye can actually provide a valuable biomarker to track the brain deterioration during the stages of AD [175,176,177]. In this connection our data, showing that 12A12mAb treatment in vivo enhances the structural plasticity and permits a functional recovery of neural circuits by normalizing the dysregulation of actin dynamics in the primary visual cortex, are much more consistent with the hypothesis that the neurotoxic N-truncated tau in the Tg2576 AD mouse model locally engages signaling pathways for dendritic spine loss by inducing the ADF/cofilin dephosphorylation, as previously reported in their hippocampi [42].

## 5. Conclusions

In conclusion, this study demonstrates that the chronic accumulation of pathogenic truncated NH_2_htau fragments along the visual pathway, including the V1 area, of Tg2576 AD mice leads to functional alterations in their visual acuity behaviour in a way that positively responds to beneficial immunization with 12A12mAb. Our data might have broader implications in mitigating the impact of tau pathology on vision deficits in several visual disorders by offering an innovative, alternative approach for the cure of these devastating illnesses that hardly compromise the life quality of patients affected by AD and, possibly, from other human tauopathies.

## Data Availability

All the data used and/or analyzed for the current study is contained in the article. All other datasets are available from the corresponding author upon reasonable request.

## Supplementary Materials

The following supporting information can be downloaded at: https://www.mdpi.com/article/10.3390/pharmaceutics15020509/s1, Figure S1: The steady-state expression level of APP/Aβ is reduced in V1 synapses from in Tg2576 AD mice following 12A12mAb treatment; Figure S2: Visual Acuity performance of all three experimental groups progressively decreased on consecutive P1, P2, P3 and P4 “testing phases”; Figure S3: No overt neuron loss and gross histopathological alterations are observed in primary visual cortex from 6-month-old Tg2576 AD mice.

## References

[1] Murphy C. (2019). Olfactory and other sensory impairments in Alzheimer disease. Nat. Rev. Neurol..

[2] Gupta V.B., Chitranshi N., den Haan J., Mirzaei M., You Y., Lim J.K., Basavarajappa D., Godinez A., Di Angelantonio S., Sachdev P. (2021). Retinal changes in Alzheimer’s disease- integrated prospects of imaging, functional and molecular advances. Prog. Retin. Eye Res..

[3] Mirzaei N., Shi H., Oviatt M., Doustar J., Rentsendorj A., Fuchs D.T., Sheyn J., Black K.L., Koronyo Y., Koronyo-Hamaoui M. (2020). Alzheimer’s Retinopathy: Seeing Disease in the Eyes. Front. Neurosci..

[4] Zhang J., Shi L., Shen Y. (2022). The retina: A window in which to view the pathogenesis of Alzheimer’s disease. Ageing Res. Rev..

[5] Shah T.M., Gupta S.M., Chatterjee P., Campbell M., Martins R.N. (2017). Beta-amyloid sequelae in the eye: A critical review on its diagnostic significance and clinical relevance in Alzheimer’s disease. Mol. Psychiatry.

[6] Marchesi N., Fahmideh F., Boschi F., Pascale A., Barbieri A. (2021). Ocular Neurodegenerative Diseases: Interconnection between Retina and Cortical Areas. Cells.

[7] Guo L., Ravindran N., Shamsher E., Hill D., Cordeiro M.F. (2021). Retinal Changes in Transgenic Mouse Models of Alzheimer’s Disease. Curr. Alzheimer Res..

[8] Chiquita S., Rodrigues-Neves A.C., Baptista F.I., Carecho R., Moreira P.I., Castelo-Branco M., Ambrósio A.F. (2019). The Retina as a Window or Mirror of the Brain Changes Detected in Alzheimer’s Disease: Critical Aspects to Unravel. Mol. Neurobiol..

[9] London A., Benhar I., Schwartz M. (2013). The retina as a window to the brain: From eye research to CNS disorders. Nat. Rev. Neurol..

[10] Chiasseu M., Alarcon-Martinez L., Belforte N., Quintero H., Dotigny F., Destroismaisons L., Velde C.V., Panayi F., Louis C., Di Polo A. (2017). Tau accumulation in the retina promotes early neuronal dysfunction and precedes brain pathology in a mouse model of Alzheimer’s disease. Mol. Neurodegener..

[11] Criscuolo C., Cerri E., Fabiani C., Capsoni S., Cattaneo A., Domenici L. (2018). The retina as a window to early dysfunctions of Alzheimer’s disease following studies with a 5xFAD mouse model. Neurobiol. Aging.

[12] Latina V., Giacovazzo G., Cordella F., Balzamino B.O., Micera A., Varano M., Marchetti C., Malerba F., Florio R., Ercole B.B. (2021). Systemic delivery of a specific antibody targeting the pathological N-terminal truncated tau peptide reduces retinal degeneration in a mouse model of Alzheimer’s Disease. Acta Neuropathol. Commun..

[13] Cormack F.K., Tovee M., Ballard C. (2000). Contrast sensitivity and visual acuity in patients with Alzheimer’s disease. Int. J. Geriatr. Psychiatry.

[14] Albers M.W., Gilmore G.C., Kaye J., Murphy C., Wingfield A., Bennett D.A., Boxer A.L., Buchman A.S., Cruickshanks K.J., Devanand D.P. (2015). At the interface of sensory and motor dysfunctions and Alzheimer’s disease. Alzheimer’s Dement..

[15] Armstrong R., Kergoat H. (2015). Oculo-visual changes and clinical considerations affecting older patients with dementia. Ophthalmic Physiol. Opt..

[16] Kusne Y., Wolf A.B., Townley K., Conway M., Peyman G.A. (2017). Visual system manifestations of Alzheimer’s disease. Acta Ophthalmol..

[17] Glosser G., Gallo J., Duda N., de Vries J.J., Clark C.M., Grossman M. (2002). Visual perceptual functions predict instrumental activities of daily living in patients with dementia. Neuropsychiatry Neuropsychol. Behav. Neurol..

[18] Rizzo M., Anderson S.W., Dawson J., Nawrot M. (2000). Vision and cognition in Alzheimer’s disease. Neuropsychologia.

[19] Tippett L.J., Blackwood K., Farah M.J. (2003). Visual object and face processing in mild-to-moderate Alzheimer’s disease: From segmentation to imagination. Neuropsychologia.

[20] Ngolab J., Honma P., Rissman R.A. (2019). Reflections on the Utility of the Retina as a Biomarker for Alzheimer’s Disease: A Literature Review. Neurol. Ther..

[21] den Haan J., Morrema T.H.J., Verbraak F.D., de Boer J.F., Scheltens P., Rozemuller A.J., Bergen A.A.B., Bouwman F.H., Hoozemans J.J. (2018). Amyloid-beta and phosphorylated tau in post-mortem Alzheimer’s disease retinas. Acta Neuropathol. Commun..

[22] Blazes M., Lee C.S. (2021). Understanding the Brain through Aging Eyes. Adv. Geriatr. Med. Res..

[23] Hart N.J., Koronyo Y., Black K.L., Koronyo-Hamaoui M. (2016). Ocular indicators of Alzheimer’s: Exploring disease in the retina. Acta Neuropathol..

[24] Majeed A., Marwick B., Yu H., Fadavi H., Tavakoli M. (2021). Ophthalmic Biomarkers for Alzheimer’s Disease: A Review. Front. Aging Neurosci..

[25] Ge Y.J., Xu W., Ou Y.N., Qu Y., Ma Y.H., Huang Y.Y., Shen X.N., Chen S.D., Tan L., Zhao Q.H. (2021). Retinal biomarkers in Alzheimer’s disease and mild cognitive impairment: A systematic review and meta-analysis. Ageing Res. Rev..

[26] Liao C., Xu J., Chen Y., Ip N.Y. (2021). Retinal Dysfunction in Alzheimer’s Disease and Implications for Biomarkers. Biomolecules.

[27] Binetti G., Cappa S.F., Magni E., Padovani A., Bianchetti A., Trabucchi M. (1998). Visual and spatial perception in the early phase of Alzheimer’s disease. Neuropsychology.

[28] Alescio-Lautier B., Michel B.F., Herrera C., Elahmadi A., Chambon C., Touzet C., Paban V. (2007). Visual and visuospatial short-term memory in mild cognitive impairment and Alzheimer disease: Role of attention. Neuropsychologia.

[29] Tales A., Haworth J., Nelson S., Snowden R.J., Wilcock G. (2005). Abnormal visual search in mild cognitive impairment and Alzheimer’s disease. Neurocase.

[30] Lemos R., Santana I., Caetano G., Bernardino I., Morais R., Farivar R., Castelo-Branco M. (2016). Three-Dimensional Face Recognition in Mild Cognitive Impairment: A Psychophysical and Structural MR Study. J. Int. Neuropsychol. Soc..

[31] Graewe B., Lemos R., Ferreira C., Santana I., Farivar R., De Weerd P., Castelo-Branco M. (2013). Impaired processing of 3D motion-defined faces in mild cognitive impairment and healthy aging: An fMRI study. Cereb. Cortex.

[32] Marquié M., Castilla-Martí M., Valero S., Martínez J., Sánchez D., Hernández I., Rosende-Roca M., Vargas L., Mauleón A., Rodríguez-Gómez O. (2019). Visual impairment in aging and cognitive decline: Experience in a Memory Clinic. Sci. Rep..

[33] Tong F. (2003). Primary visual cortex and visual awareness. Nat. Rev. Neurosci..

[34] Ikonomovic M.D., Mufson E.J., Wuu J., Bennett D.A., DeKosky S.T. (2005). Reduction of choline acetyltransferase activity in primary visual cortex in mild to moderate Alzheimer’s disease. Arch. Neurol..

[35] Javaid F.Z., Brenton J., Guo L., Cordeiro M.F. (2016). Visual and Ocular Manifestations of Alzheimer’s Disease and Their Use as Biomarkers for Diagnosis and Progression. Front. Neurol..

[36] Gămănuţ R., Shimaoka D. (2021). Anatomical and functional connectomes underlying hierarchical visual processing in mouse visual system. Brain Struct. Funct..

[37] Miller M.W., Vogt B.A. (1984). Direct connections of rat visual cortex with sensory, motor, and association cortices. J. Comp. Neurol..

[38] Vaudano E., Legg C.R., Glickstein M. (1991). Afferent and Efferent Connections of Temporal Association Cortex in the Rat: A Horseradish Peroxidase Study. Eur. J. Neurosci..

[39] Lavenex P., Amaral D.G. (2000). Hippocampal-neocortical interaction: A hierarchy of associativity. Hippocampus.

[40] Furtak S.C., Wei S.M., Agster K.L., Burwell R.D. (2007). Functional neuroanatomy of the parahippocampal region in the rat: The perirhinal and postrhinal cortices. Hippocampus.

[41] Haggerty D.C., Ji D. (2015). Activities of visual cortical and hippocampal neurons co-fluctuate in freely moving rats during spatial behavior. Elife.

[42] Corsetti V., Borreca A., Latina V., Giacovazzo G., Pignataro A., Krashia P., Natale F., Cocco S., Rinaudo M., Malerba F. (2020). Passive immunotherapy for N-truncated tau ameliorates the cognitive deficits in two mouse Alzheimer’s disease models. Brain Commun..

[43] Hsiao K., Chapman P., Nilsen S., Eckman C., Harigaya Y., Younkin S., Yang F., Cole G. (1996). Correlative memory deficits, Abeta elevation, and amyloid plaques in transgenic mice. Science.

[44] Sasaguri H., Nilsson P., Hashimoto S., Nagata K., Saito T., De Strooper B., Hardy J., Vassar R., Winblad B., Saido T.C. (2017). APP mouse models for Alzheimer’s disease preclinical studies. EMBO J..

[45] Yassine N., Lazaris A., Dorner-Ciossek C., Després O., Meyer L., Maitre M., Mensah-Nyagan A.G., Cassel J.C., Mathis C. (2013). Detecting spatial memory deficits beyond blindness in tg2576 Alzheimer mice. Neurobiol. Aging.

[46] Castillo-Carranza D.L., Guerrero-Muñoz M.J., Sengupta U., Hernandez C., Barrett A.D., Dineley K., Kayed R. (2015). Tau immunotherapy modulates both pathological tau and upstream amyloid pathology in an Alzheimer’s disease mouse model. J. Neurosci..

[47] Schmolesky M., Kolb H., Fernandez E., Nelson R. (1995). The Primary Visual Cortex. Webvision: The Organization of the Retina and Visual System [Internet].

[48] Paxinos G., Franklin K.B.J. (2001). The Mouse Brain in Stereotaxic Coordinates.

[49] Gao M., Sossa K., Song L., Errington L., Cummings L., Hwang H., Kuhl D., Worley P., Lee H.K. (2010). A specific requirement of Arc/Arg3.1 for visual experience-induced homeostatic synaptic plasticity in mouse primary visual cortex. J. Neurosci..

[50] El-Boustani S., Ip J.P.K., Breton-Provencher V., Knott G.W., Okuno H., Bito H., Sur M. (2018). Locally coordinated synaptic plasticity of visual cortex neurons in vivo. Science.

[51] D’Amelio M., Cavallucci V., Middei S., Marchetti C., Pacioni S., Ferri A., Diamantini A., De Zio D., Carrara P., Battistini L. (2011). Caspase-3 triggers early synaptic dysfunction in a mouse model of Alzheimer’s disease. Nat. Neurosci..

[52] Schägger H., von Jagow G. (1987). Tricine-sodium dodecyl sulfate-polyacrylamide gel electrophoresis for the separation of proteins in the range from 1 to 100 kDa. Anal. Biochem..

[53] Amadoro G., Corsetti V., Atlante A., Florenzano F., Capsoni S., Bussani R., Mercanti D., Calissano P. (2012). Interaction between NH(2)-tau fragment and Aβ in Alzheimer’s disease mitochondria contributes to the synaptic deterioration. Neurobiol. Aging.

[54] Corsetti V., Amadoro G., Gentile A., Capsoni S., Ciotti M.T., Cencioni M.T., Atlante A., Canu N., Rohn T.T., Cattaneo A. (2008). Identification of a caspase-derived N-terminal tau fragment in cellular and animal Alzheimer’s disease models. Mol. Cell. Neurosci..

[55] Rohn T.T., Rissman R.A., Davis M.C., Kim Y.E., Cotman C.W., Head E. (2002). Caspase-9 activation and caspase cleavage of tau in the Alzheimer’s disease brain. Neurobiol. Dis..

[56] Pignataro A., Meli G., Pagano R., Fontebasso V., Battistella R., Conforto G., Ammassari-Teule M., Middei S. (2019). Activity-Induced Amyloid-β Oligomers Drive Compensatory Synaptic Rearrangements in Brain Circuits Controlling Memory of Presymptomatic Alzheimer’s Disease Mice. Biol. Psychiatry.

[57] Rosoklija G.B., Petrushevski V.M., Stankov A., Dika A., Jakovski Z., Pavlovski G., Davcheva N., Lipkin R., Schnieder T., Scobie K. (2014). Reliable and durable Golgi staining of brain tissue from human autopsies and experimental animals. J. Neurosci. Methods.

[58] Risher W.C., Ustunkaya T., Alvarado J.S., Eroglu C. (2014). Rapid Golgi analysis method for efficient and unbiased classification of dendritic spines. PLoS ONE.

[59] Gibb R., Kolb B. (1998). A method for vibratome sectioning of Golgi-Cox stained whole rat brain. J. Neurosci. Methods.

[60] Pignataro A., Borreca A., Ammassari-Teule M., Middei S. (2015). CREB Regulates Experience-Dependent Spine Formation and Enlargement in Mouse Barrel Cortex. Neural Plast..

[61] Prusky G.T., West P.W., Douglas R.M. (2000). Behavioral assessment of visual acuity in mice and rats. Vis. Res..

[62] Robinson L., Bridge H., Riedel G. (2001). Visual discrimination learning in the water maze: A novel test for visual acuity. Behav. Brain Res..

[63] Robinson L., Harbaran D., Riedel G. (2004). Visual acuity in the water maze: Sensitivity to muscarinic receptor blockade in rats and mice. Behav. Brain Res..

[64] Marrocco E., Indrieri A., Esposito F., Tarallo V., Carboncino A., Alvino F.G., De Falco S., Franco B., De Risi M., De Leonibus E. (2020). α-synuclein overexpression in the retina leads to vision impairment and degeneration of dopaminergic amacrine cells. Sci. Rep..

[65] Crijns E., Op de Beeck H. (2019). The Visual Acuity of Rats in Touchscreen Setups. Vision.

[66] Himmelhan D.K., Rawashdeh O., Oelschläger H.H.A. (2018). Early postnatal development of the visual cortex in mice with retinal degeneration. Mech. Dev..

[67] Palomero-Gallagher N., Zilles K., Paxinos I.G. (2004). The Rat Nervous System.

[68] Paxinos G., Franklin K.B.J. (2012). Paxinos and Franklin’s the Mouse Brain in Stereotaxic Coordinates.

[69] Allen Institute for Brain Science (2014). Allen’s Mouse Brain Atlas—The Brain Explorer^®^ 2.

[70] Shihan M.H., Novo S.G., Le Marchand S.J., Wang Y., Duncan M.K. (2021). A simple method for quantitating confocal fluorescent images. Biochem. Biophys. Rep..

[71] Morrissette D.A., Parachikova A., Green K.N., LaFerla F.M. (2009). Relevance of transgenic mouse models to human Alzheimer disease. J. Biol. Chem..

[72] Priebe N.J., McGee A.W. (2014). Mouse vision as a gateway for understanding how experience shapes neural circuits. Front. Neural Circuits.

[73] Niell C.M., Stryker M.P. (2008). Highly selective receptive fields in mouse visual cortex. J. Neurosci..

[74] Iacaruso M.F., Gasler I.T., Hofer S.B. (2017). Synaptic organization of visual space in primary visual cortex. Nature.

[75] Asavapanumas N., Brawek B., Martus P., Garaschuk O. (2019). Role of intracellular Ca^2+^ stores for an impairment of visual processing in a mouse model of Alzheimer’s disease. Neurobiol. Dis..

[76] Jorge L., Canário N., Martins R., Santiago B., Santana I., Quental H., Ambrósio F., Bernardes R., Castelo-Branco M. (2020). The Retinal Inner Plexiform Synaptic Layer Mirrors Grey Matter Thickness of Primary Visual Cortex with Increased Amyloid β Load in Early Alzheimer’s Disease. Neural Plast..

[77] Jorge L., Canário N., Quental H., Bernardes R., Castelo-Branco M. (2020). Is the Retina a Mirror of the Aging Brain? Aging of Neural Retina Layers and Primary Visual Cortex Across the Lifespan. Front. Aging Neurosci..

[78] Rodriguez L., Joly S., Zine-Eddine F., Mdzomba J.B., Pernet V. (2020). Tau modulates visual plasticity in adult and old mice. Neurobiol. Aging.

[79] Leuba G., Kraftsik R. (1994). Visual cortex in Alzheimer’s disease: Occurrence of neuronal death and glial proliferation, and correlation with pathological hallmarks. Neurobiol. Aging.

[80] Ho W., Leung Y., Tsang A.W., So K.F., Chiu K., Chang R.C. (2012). Review: Tauopathy in the retina and optic nerve: Does it shadow pathological changes in the brain?. Mol. Vis..

[81] Rahimi J., Milenkovic I., Kovacs G.G. (2015). Patterns of Tau and α-Synuclein Pathology in the Visual System. J. Park. Dis..

[82] Arouche-Delaperche L., Cadoni S., Joffrois C., Labernede G., Valet M., César Q., Dégardin J., Girardon S., Gabriel C., Krantic S. (2023). Dysfunction of the glutamatergic photoreceptor synapse in the P301S mouse model of tauopathy. Acta Neuropathol Commun..

[83] Cerquera-Jaramillo M.A., Nava-Mesa M.O., González-Reyes R.E., Tellez-Conti C., de-la-Torre A. (2018). Visual Features in Alzheimer’s Disease: From Basic Mechanisms to Clinical Overview. Neural Plast..

[84] Paik J.S., Ha M., Jung Y.H., Kim G.H., Han K.D., Kim H.S., Lim D.H., Na K.S. (2020). Low vision and the risk of dementia: A nationwide population-based cohort study. Sci. Rep..

[85] Elyashiv S.M., Shabtai E.L., Belkin M. (2014). Correlation between visual acuity and cognitive functions. Br. J. Ophthalmol..

[86] Liebscher S., Keller G.B., Goltstein P.M., Bonhoeffer T., Hübener M. (2016). Selective Persistence of Sensorimotor Mismatch Signals in Visual Cortex of Behaving Alzheimer’s Disease mice. Curr. Biol..

[87] Grienberger C., Rochefort N.L., Adelsberger H., Henning H.A., Hill D.N., Reichwald J., Staufenbiel M., Konnerth A. (2012). Staged decline of neuronal function in vivo in an animal model of Alzheimer’s disease. Nat. Commun..

[88] Papanikolaou A., Rodrigues F.R., Holeniewska J., Phillips K.G., Saleem A.B., Solomon S.G. (2022). Plasticity in visual cortex is disrupted in a mouse model of tauopathy. Commun. Biol..

[89] Stover K.R., Brown R.E. (2012). Age-related changes in visual acuity, learning and memory in the APPswe/PS1dE9 mouse model of Alzheimer’s disease. Behav. Brain Res..

[90] Vit J.P., Fuchs D.T., Angel A., Levy A., Lamensdorf I., Black K.L., Koronyo Y., Koronyo-Hamaoui M. (2021). Color and contrast vision in mouse models of aging and Alzheimer’s disease using a novel visual-stimuli four-arm maze. Sci. Rep..

[91] Turner K.M., Simpson C.G., Burne T.H.J. (2017). Touchscreen-based Visual Discrimination and Reversal Tasks for Mice to Test Cognitive Flexibility. Bio-Protocol.

[92] Storchi R., Rodgers J., Gracey M., Martial F.P., Wynne J., Ryan S., Twining C.J., Cootes T.F., Killick R., Lucas R.J. (2019). Measuring vision using innate behaviours in mice with intact and impaired retina function. Sci. Rep..

[93] Nikolaienko O., Patil S., Eriksen M.S., Bramham C.R. (2018). Arc protein: A flexible hub for synaptic plasticity and cognition. Semin. Cell Dev. Biol..

[94] Tagawa Y., Kanold P.O., Majdan M., Shatz C.J. (2005). Multiple periods of functional ocular dominance plasticity in mouse visual cortex. Nat. Neurosci..

[95] Jenks K.R., Kim T., Pastuzyn E.D., Okuno H., Taibi A.V., Bito H., Bear M.F., Shepherd J.D. (2017). Arc restores juvenile plasticity in adult mouse visual cortex. Proc. Natl. Acad. Sci. USA.

[96] William C.M., Stern M.A., Pei X., Saqran L., Ramani M., Frosch M.P., Hyman B.T. (2021). Impairment of visual cortical plasticity by amyloid-beta species. Neurobiol. Dis..

[97] Rudinskiy N., Hawkes J.M., Wegmann S., Kuchibhotla K.V., Muzikansky A., Betensky R.A., Spires-Jones T.L., Hyman B.T. (2014). Tau pathology does not affect experience-driven single-neuron and network-wide Arc/Arg3.1 responses. Acta Neuropathol. Commun..

[98] Rudinskiy N., Hawkes J.M., Betensky R.A., Eguchi M., Yamaguchi S., Spires-Jones T.L., Hyman B.T. (2012). Orchestrated experience-driven Arc responses are disrupted in a mouse model of Alzheimer’s disease. Nat. Neurosci..

[99] Dickey C.A., Loring J.F., Montgomery J., Gordon M.N., Eastman P.S., Morgan D. (2003). Selectively reduced expression of synaptic plasticity-related genes in amyloid precursor protein + presenilin-1 transgenic mice. J. Neurosci..

[100] Dickey C.A., Gordon M.N., Mason J.E., Wilson N.J., Diamond D.M., Guzowski J.F., Morgan D. (2004). Amyloid suppresses induction of genes critical for memory consolidation in APP + PS1 transgenic mice. J. Neurochem..

[101] Di Cristo G., Berardi N., Cancedda L., Pizzorusso T., Putignano E., Ratto G.M., Maffei L. (2001). Requirement of ERK activation for visual cortical plasticity. Science.

[102] Caleo M., Restani L., Gianfranceschi L., Costantin L., Rossi C., Rossetto O., Montecucco C., Maffei L. (2007). Transient synaptic silencing of developing striate cortex has persistent effects on visual function and plasticity. J. Neurosci..

[103] Fox L.M., William C.M., Adamowicz D.H., Pitstick R., Carlson G.A., Spires-Jones T.L., Hyman B.T. (2011). Soluble tau species, not neurofibrillary aggregates, disrupt neural system integration in a tau transgenic model. J. Neuropathol. Exp. Neurol..

[104] Morin J.P., Cerón-Solano G., Velázquez-Campos G., Pacheco-López G., Bermúdez-Rattoni F., Díaz-Cintra S. (2016). Spatial Memory Impairment is Associated with Intraneural Amyloid-β Immunoreactivity and Dysfunctional Arc Expression in the Hippocampal-CA3 Region of a Transgenic Mouse Model of Alzheimer’s Disease. J. Alzheimer’s Dis..

[105] Wang K.H., Majewska A., Schummers J., Farley B., Hu C., Sur M., Tonegawa S. (2006). In vivo two-photon imaging reveals a role of arc in enhancing orientation specificity in visual cortex. Cell.

[106] Ramirez-Amaya V., Vazdarjanova A., Mikhael D., Rosi S., Worley P.F., Barnes C.A. (2005). Spatial exploration-induced Arc mRNA and protein expression: Evidence for selective, network-specific reactivation. J. Neurosci..

[107] Lind D., Franken S., Kappler J., Jankowski J., Schilling K. (2005). Characterization of the neuronal marker NeuN as a multiply phosphorylated antigen with discrete subcellular localization. J. Neurosci. Res..

[108] Wallace W., Bear M.F. (2004). A morphological correlate of synaptic scaling in visual cortex. J. Neurosci..

[109] Tropea D., Majewska A.K., Garcia R., Sur M. (2010). Structural dynamics of synapses in vivo correlate with functional changes during experience-dependent plasticity in visual cortex. J. Neurosci..

[110] Chen J.L., Villa K.L., Cha J.W., So P.T.C., Kubota Y., Nedivi E. (2012). Clustered Dynamics of Inhibitory Synapses and Dendritic Spines in the Adult Neocortex. Neuron.

[111] Yu H., Majewska A.K., Sur M. (2011). Rapid experience-dependent plasticity of synapse function and structure in ferret visual cortex in vivo. Proc. Natl. Acad. Sci. USA.

[112] Zablah B.Y., Merovitch N., Jia Z. (2020). The Role of ADF/Cofilin in Synaptic Physiology and Alzheimer’s Disease. Front. Cell Dev. Biol..

[113] Renouard L., Bridi M.C.D., Coleman T., Arckens L., Frank M.G. (2018). Anatomical correlates of rapid eye movement sleep-dependent plasticity in the developing cortex. Sleep.

[114] Dahlhaus M., Li K.W., Van Der Schors R.C., Saiepour M.H., Van Nierop P., Heimel J.A., Hermans J.M., Loos M., Smit A.B., Levelt C.N. (2011). The synaptic proteome during development and plasticity of the mouse visual cortex. Mol. Cell. Proteom..

[115] Bornia N., Taboada A., Dapueto A., Rossi F.M. (2020). Identification of cofilin 1 as a candidate protein associated to mouse visual cortex plasticity. Neurosci. Lett..

[116] Levine N.D., Rademacher D.J., Collier T.J., O’Malley J.A., Kells A.P., San Sebastian W., Bankiewicz K.S., Steece-Collier K. (2013). Advances in Thin Tissue Golgi-Cox Impregnation: Fast, Reliable Methods for Multi-Assay Analyses in Rodent and Non-human Primate Brain. J. Neurosci. Methods.

[117] Lanz T.A., Carter D.B., Merchant K.M. (2003). Dendritic spine loss in the hippocampus of young PDAPP and Tg2576 mice and its prevention by the ApoE2 genotype. Neurobiol. Dis..

[118] Jacobsen J.S., Wu C.C., Redwine J.M., Comery T.A., Arias R., Bowlby M., Martone R., Morrison J.H., Pangalos M.N., Reinhart P.H. (2006). Early-onset behavioral and synaptic deficits in a mouse model of Alzheimer’s disease. Proc. Natl. Acad. Sci. USA.

[119] Rahman T., Davies D.S., Tannenberg R.K., Fok S., Shepherd C., Dodd P.R., Cullen K.M., Goldsbury C. (2014). Cofilin rods and aggregates concur with tau pathology and the development of Alzheimer’s disease. J. Alzheimer’s Dis..

[120] Bamburg J.R., Bernstein B.W. (2016). Actin dynamics and cofilin-actin rods in alzheimer disease. Cytoskeleton.

[121] Kang D.E., Woo J.A. (2019). Cofilin, a Master Node Regulating Cytoskeletal Pathogenesis in Alzheimer’s Disease. J. Alzheimer’s Dis..

[122] Davis R.C., Marsden I.T., Maloney M.T., Minamide L.S., Podlisny M., Selkoe D.J., Bamburg J.R. (2011). Amyloid beta dimers/trimers potently induce cofilin-actin rods that are inhibited by maintaining cofilin-phosphorylation. Mol. Neurodegener..

[123] Mendoza-Naranjo A., Contreras-Vallejos E., Henriquez D.R., Otth C., Bamburg J.R., Maccioni R.B., Gonzalez-Billault C. (2012). Fibrillar amyloid-β1–42 modifies actin organization affecting the cofilin phosphorylation state: A role for Rac1/cdc42 effector proteins and the slingshot phosphatase. J. Alzheimer’s Dis..

[124] Barone E., Mosser S., Fraering P.C. (2014). Inactivation of brain Cofilin-1 by age, Alzheimer’s disease and γ-secretase. Biochim. Biophys. Acta.

[125] Norton D.J., Parra M.A., Sperling R.A., Baena A., Guzman-Velez E., Jin D.S., Andrea N., Khang J., Schultz A., Rentz D.M. (2020). Visual short-term memory relates to tau and amyloid burdens in preclinical autosomal dominant Alzheimer’s disease. Alzheimer’s Res. Ther..

[126] Bocanegra Y., Fox-Fuller J.T., Baena A., Guzmán-Vélez E., Vila-Castelar C., Martínez J., Torrico-Teave H., Lopera F., Quiroz Y.T. (2021). Association Between Visual Memory and In Vivo Amyloid and Tau Pathology in Preclinical Autosomal Dominant Alzheimer’s Disease. J. Int. Neuropsychol. Soc..

[127] Putcha D., Brickhouse M., Touroutoglou A., Collins J.A., Quimby M., Wong B., Eldaief M., Schultz A., El Fakhri G., Johnson K. (2019). Visual cognition in non-amnestic Alzheimer’s disease: Relations to tau, amyloid, and cortical atrophy. Neuroimage Clin..

[128] Gilmore G.C., Cronin-Golomb A., Neargarder S.A., Morrison S.R. (2005). Enhanced stimulus contrast normalizes visual processing of rapidly presented letters in Alzheimer’s disease. Vis. Res..

[129] Kavcic V., Duffy C.J. (2003). Attentional dynamics and visual perception: Mechanisms of spatial disorientation in Alzheimer’s disease. Brain.

[130] Schlotterer G., Moscovitch M., Crapper-McLachlan D. (1984). Visual processing deficits as assessed by spatial frequency contrast sensitivity and backward masking in normal ageing and Alzheimer’s disease. Brain.

[131] Bonney K.R., Almeida O.P., Flicker L., Davies S., Clarnette R., Anderson M., Lautenschlager N.T. (2006). Inspection time in non-demented older adults with mild cognitive impairment. Neuropsychologia.

[132] Perry R.J., Hodges J.R. (2003). Dissociation between top-down attentional control and the time course of visual attention as measured by attentional dwell time in patients with mild cognitive impairment. Eur. J. Neurosci..

[133] Cunha J.P., Moura-Coelho N., Proença R.P., Dias-Santos A., Ferreira J., Louro C., Castanheira-Dinis A. (2016). Alzheimer’s disease: A review of its visual system neuropathology. Optical coherence tomography—A potential role as a study tool in vivo. Graefes Arch. Clin. Exp. Ophthalmol..

[134] Kirby E., Bandelow S., Hogervorst E. (2010). Visual impairment in Alzheimer’s disease: A critical review. J. Alzheimer’s Dis..

[135] Chang L.Y.L., Lowe J., Ardiles A., Lim J., Grey A.C., Robertson K., Danesh-Meyer H., Palacios A.G., Acosta M.L. (2014). Alzheimer’s disease in the human eye. Clinical tests that identify ocular and visual information processing deficit as biomarkers. Alzheimers Dement..

[136] Huang J., Beach P., Bozoki A., Zhu D.C. (2021). Alzheimer’s Disease Progressively Reduces Visual Functional Network Connectivity. J. Alzheimer’s Dis. Rep..

[137] Huang J., Beach P., Bozoki A., Zhu D.C. (2020). Alzheimer’s Disease Progressively Alters the Face-Evoked Visual-Processing Network. J. Alzheimer’s Dis..

[138] Rehan S., Giroud N., Al-Yawer F., Wittich W., Phillips N. (2021). Visual Performance and Cortical Atrophy in Vision-Related Brain Regions Differ Between Older Adults with (or at Risk for) Alzheimer’s Disease. J. Alzheimer’s Dis..

[139] Chen C., Ma X., Wei J., Shakir N., Zhang J.K., Zhang L., Nehme A., Cui Y., Ferguson D., Bai F. (2022). Early impairment of cortical circuit plasticity and connectivity in the 5XFAD Alzheimer’s disease mouse model. Transl. Psychiatry.

[140] O’Leary T.P., Brown R.E. (2009). Visuo-spatial learning and memory deficits on the Barnes maze in the 16-month-old APPswe/PS1dE9 mouse model of Alzheimer’s disease. Behav. Brain Res..

[141] Wong A.A., Brown R.E. (2006). Visual detection, pattern discrimination and visual acuity in 14 strains of mice. Genes Brain Behav..

[142] Brown R.E., Wong A.A. (2007). The influence of visual ability on learning and memory performance in 13 strains of mice. Learn. Mem..

[143] Buscher N., van Dorsselaer P., Steckler T., Talpos J.C. (2017). Evaluating aged mice in three touchscreen tests that differ in visual demands: Impaired cognitive function and impaired visual abilities. Behav. Brain Res..

[144] Poort J., Khan A.G., Pachitariu M., Nemri A., Orsolic I., Krupic J., Bauza M., Sahani M., Keller G.B., Mrsic-Flogel T.D. (2015). Learning Enhances Sensory and Multiple Non-sensory Representations in Primary Visual Cortex. Neuron.

[145] Goard M.J., Pho G.N., Woodson J., Sur M. (2016). Distinct roles of visual, parietal, and frontal motor cortices in memory-guided sensorimotor decisions. Elife.

[146] Resulaj A., Ruediger S., Olsen S.R., Scanziani M. (2018). First spikes in visual cortex enable perceptual discrimination. Elife.

[147] Wolf A., Bauer B., Abner E.L., Ashkenazy-Frolinger T., Hartz A.M. (2016). A Comprehensive Behavioral Test Battery to Assess Learning and Memory in 129S6/Tg2576 Mice. PLoS ONE.

[148] Ognibene E., Middei S., Daniele S., Adriani W., Ghirardi O., Caprioli A., Laviola G. (2005). Aspects of spatial memory and behavioral disinhibition in Tg2576 transgenic mice as a model of Alzheimer’s disease. Behav. Brain Res..

[149] Lalonde R., Lews T.L., Strazielle C., Kim H., Fukuchi K. (2003). Transgenic mice expressing the betaAPP695SWE mutation: Effects on exploratory activity, anxiety, and motor coordination. Brain Res..

[150] Gil-Bea F.J., Aisa B., Schliebs R., Ramírez M.J. (2007). Increase of locomotor activity underlying the behavioral disinhibition in tg2576 mice. Behav. Neurosci..

[151] Roberson E.D., Scearce-Levie K., Palop J.J., Yan F., Cheng I.H., Wu T., Gerstein H., Yu G.Q., Mucke L. (2007). Reducing endogenous tau ameliorates amyloid beta-induced deficits in an Alzheimer’s disease mouse model. Science.

[152] Thompson S.M., Berkowitz L.E., Clark B.J. (2018). Behavioral and neural subsystems of rodent exploration. Learn. Motiv..

[153] Kaneko M., Fu Y., Stryker M.P. (2017). Locomotion induces stimulus-specific response enhancement in adult visual cortex. J. Neurosci..

[154] Goldstein L.E., Muffat J.A., Cherny R.A., Moir R.D., Ericsson M.H., Huang X., Mavros C., Coccia J.A., Faget K.Y., Fitch K.A. (2003). Cytosolic beta-amyloid deposition and supranuclear cataracts in lenses from people with Alzheimer’s disease. Lancet.

[155] Blanks J.C., Schmidt S.Y., Torigoe Y., Porrello K.V., Hinton D.R., Blanks R.H. (1996). Retinal pathology in Alzheimer’s disease. II. Regional neuron loss and glial changes in GCL. Neurobiol. Aging.

[156] Armstrong R.A. (1996). Visual feld defects in Alzheimer’s disease patients may reflect differential pathology in the primary visual cortex. Optom. Vis. Sci..

[157] Hof P.R., Morrison J.H. (1990). Quantitative analysis of a vulnerable subset of pyramidal neurons in Alzheimer’s disease: II. Primary and secondary visual cortex. J. Comp. Neurol..

[158] McKee A.C., Au R., Cabral H.J., Kowall N.W., Seshadri S., Kubilus C.A., Drake J., Wolf P.A. (2006). Visual association pathology in preclinical Alzheimer disease. J. Neuropathol. Exp. Neurol..

[159] Berisha F., Feke G.T., Trempe C.L., McMeel J.W., Schepens C.L. (2007). Retinal abnormalities in early Alzheimer’s disease. Investig. Ophthalmol. Vis. Sci..

[160] Bublak P., Redel P., Sorg C., Kurz A., Förstl H., Müller H.J., Schneider W.X., Finke K. (2011). Staged decline of visual processing capacity in mild cognitive impairment and Alzheimer’s disease. Neurobiol. Aging.

[161] Braak H., Braak E., Bohl J. (1993). Staging of Alzheimer-related cortical destruction. Eur. Neurol..

[162] Lewis D.A., Campbell M.J., Terry R.D., Morrison J.H. (1987). Laminar and regional distributions of neurofibrillary tangles and neuritic plaques in Alzheimer’s disease: A quantitative study of visual and auditory cortices. J. Neurosci..

[163] Holtmaat A., Wilbrecht L., Knott G.W., Welker E., Svoboda K. (2006). Experience-dependent and cell-type-specific spine growth in the neocortex. Nature.

[164] Pelucchi S., Stringhi R., Marcello E. (2020). Dendritic Spines in Alzheimer’s Disease: How the Actin Cytoskeleton Contributes to Synaptic Failure. Int. J. Mol. Sci..

[165] Wang Q., Yuan W., Yang X., Wang Y., Li Y., Qiao H. (2020). Role of Cofilin in Alzheimer’s Disease. Front. Cell Dev. Biol..

[166] Bamburg J.R., Minamide L.S., Wiggan O., Tahtamouni L.H., Kuhn T.B. (2021). Cofilin and Actin Dynamics: Multiple Modes of Regulation and Their Impacts in Neuronal Development and Degeneration. Cells.

[167] Maloney M.T., Bamburg J.R. (2007). Cofilin-mediated neurodegeneration in Alzheimer’s disease and other amyloidopathies. Mol. Neurobiol..

[168] Rush T., Martinez-Hernandez J., Dollmeyer M., Frandemiche M.L., Borel E., Boisseau S., Jacquier-Sarlin M., Buisson A. (2018). Synaptotoxicity in Alzheimer’s Disease Involved a Dysregulation of Actin Cytoskeleton Dynamics through Cofilin 1 Phosphorylation. J. Neurosci..

[169] Kim T., Vidal G.S., Djurisic M., William C.M., Birnbaum M.E., Garcia K.C., Hyman B.T., Schatz C.J. (2013). Human LilrB2 is a β-amyloid receptor and its murine homolog PirB regulates synaptic plasticity in an Alzheimer’s model. Science.

[170] Woo J.A., Jung A.R., Lakshmana M.K., Bedrossian A., Lim Y., Bu J.H., Park S.A., Koo E.H., Mook-Jung I., Kang D.E. (2012). Pivotal role of the RanBP9-cofilin pathway in Aβ-induced apoptosis and neurodegeneration. Cell Death Differ..

[171] Woo J.A., Zhao X., Khan H., Penn C., Wang X., Joly-Amado A., Weeber E., Morgan D., Kang D.E. (2015). Slingshot-cofilin activation mediates mitochondrial and synaptic dysfunction via Aβ ligation to β1-integrin conformers. Cell Death Differ..

[172] Woo J.A., Bogges T., Uhlar C., Wang X., Khan H., Cappos G., Joly-Amado A., De Narvaez E., Majid S., Minamide L.S. (2015). RanBP9 at the intersection between cofilin and Aβ pathologies: Rescue of neurodegenerative changes by RanBP9 reduction. Cell Death Dis..

[173] Kommaddi R., Das D., Karunakaran S., Nanguneri S., Bapat D., Ray A., Shaw E., Bennett D.A., Nair D., Ravindranath V. (2018). Aβ mediates F-actin disassembly in dendritic spines leading to cognitive deficits in Alzheimer’s disease. J. Neurosci..

[174] Deng Y., Wei J., Cheng J., Zhong P., Xiong Z., Liu A., Lin L., Chen S., Yan Z. (2016). Partial Amelioration of Synaptic and Cognitive Deficits by Inhibiting Cofilin Dephosphorylation in an Animal Model of Alzheimer’s Disease. J. Alzheimer’s Dis..

[175] Williams P.A., Thirgood R.A., Oliphant H., Frizzati A., Littlewood E., Votruba M., Good M.A., Williams J., Morgan J.E. (2013). Retinal ganglion cell dendritic degeneration in a mouse model of Alzheimer’s disease. Neurobiol. Aging.

[176] Bevan R.J., Hughes T.R., Williams P.A., Good M.A., Morgan B.P., Morgan J.E. (2020). Retinal ganglion cell degeneration correlates with hippocampal spine loss in experimental Alzheimer’s disease. Acta Neuropathol. Commun..

[177] Mahajan D., Votruba M. (2017). Can the retina be used to diagnose and plot the progression of Alzheimer’s disease?. Acta Ophthalmol..

