# Supplementary material for: Immunotherapy with Cleavage-Specific 12A12mAb Reduces the Tau Cleavage in Visual Cortex and Improves Visuo-Spatial Recognition Memory in Tg2576 AD Mouse Model
Source: Pharmaceutics. 2023 Feb 3;15(2):509. doi: 10.3390/pharmaceutics15020509 (PMC9965010; doi:10.3390/pharmaceutics15020509)
Supplement: Supplementary file 1 [file pharmaceutics-15-00509-s001.zip › pharmaceutics-2143923-supplementary.pdf]

pharmaceutics

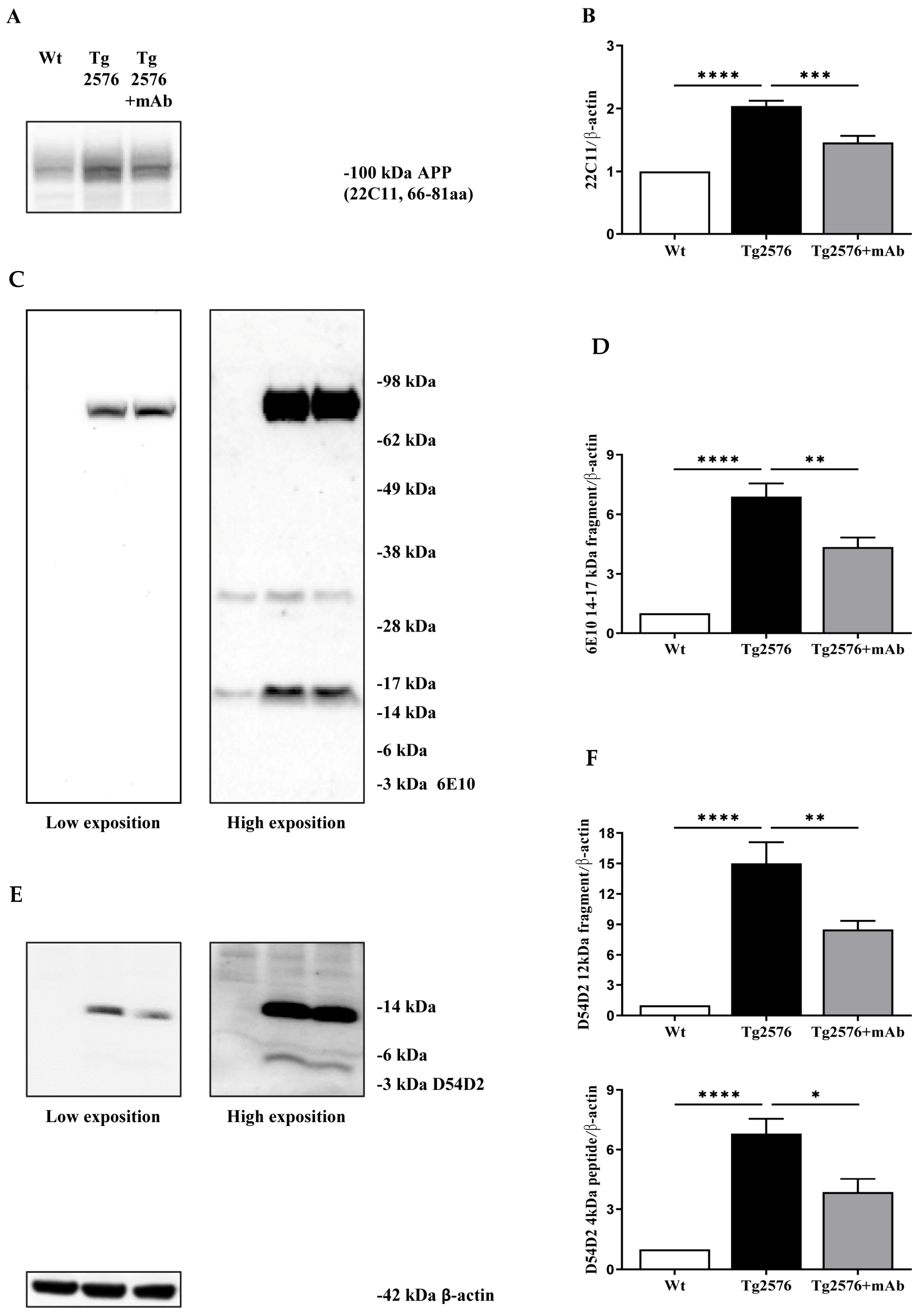

Figure S1. The steady-state expression level of APP/Aβ is reduced in V1 synapses in Tg2576 AD mice following 12A12mAb treatment. (**A**–**F**) Synaptic fractions of primary visual cortex (V1) from animals (n = 6 animals per each group, 3 males and 3 females for each experimental condition) of three experimental groups (littermate wild-type, naive/vehicle-treated Tg2576, Tg2576+mAb) were analyzed by Tris/Tricine 10–20% western blotting with antibodies reported alongside the blots (**A**,**C**,**E**). Arrows on the right side indicate the molecular weight (kDa) of bands calculated from migration of standard proteins. Semi-quantitative densitometry of the intensity signals of bands was carried out following normalization with β-actin level used as loading control (**B**,**D**,**F**). Values are from at least three independent experiments and statistically significant differences were calculated by one-way ANOVA followed by Bonferroni's post hoc test for multiple comparisons among more than two groups. $p < 0.05$ was accepted as statistically significant (* $p < 0.05$; ** $p < 0.01$; *** $p < 0.0005$; **** $p < 0.0001$).

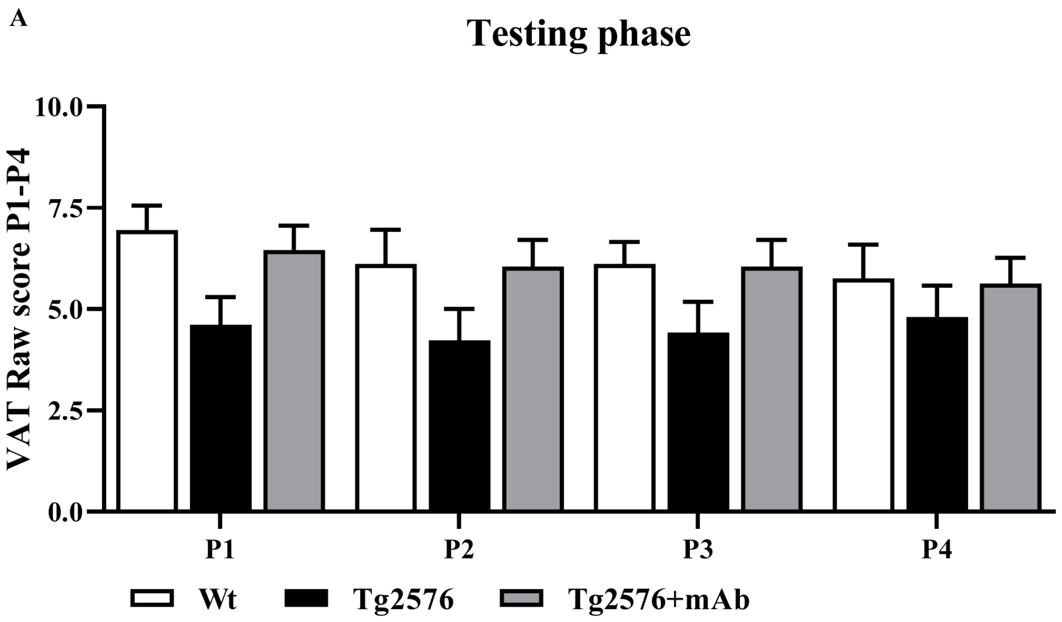

Figure S2. Visual Acuity performance of all three experimental groups progressively decreased on consecutive P1, P2, P3 and P4 "testing phases". (**A**) Animals from three experimental groups (littermate wild-type, naive/vehicle-treated Tg2576, Tg2576+mAb) were tested for their visual acuity in the Prusky's test, as described (see Materials and Methods). Histogram shows the visual acuity test "raw" score in the "testing phase" on consecutive P1, P2, P3 and P4 phases (width varies from 2 cm to 0.5 cm black). Values are from at least three independent experiments and statistically significant differences were calculated by repeated measures ANOVA followed by Fisher least significant difference (LSD) post hoc test. $p < 0.05$ was accepted as statistically significant.

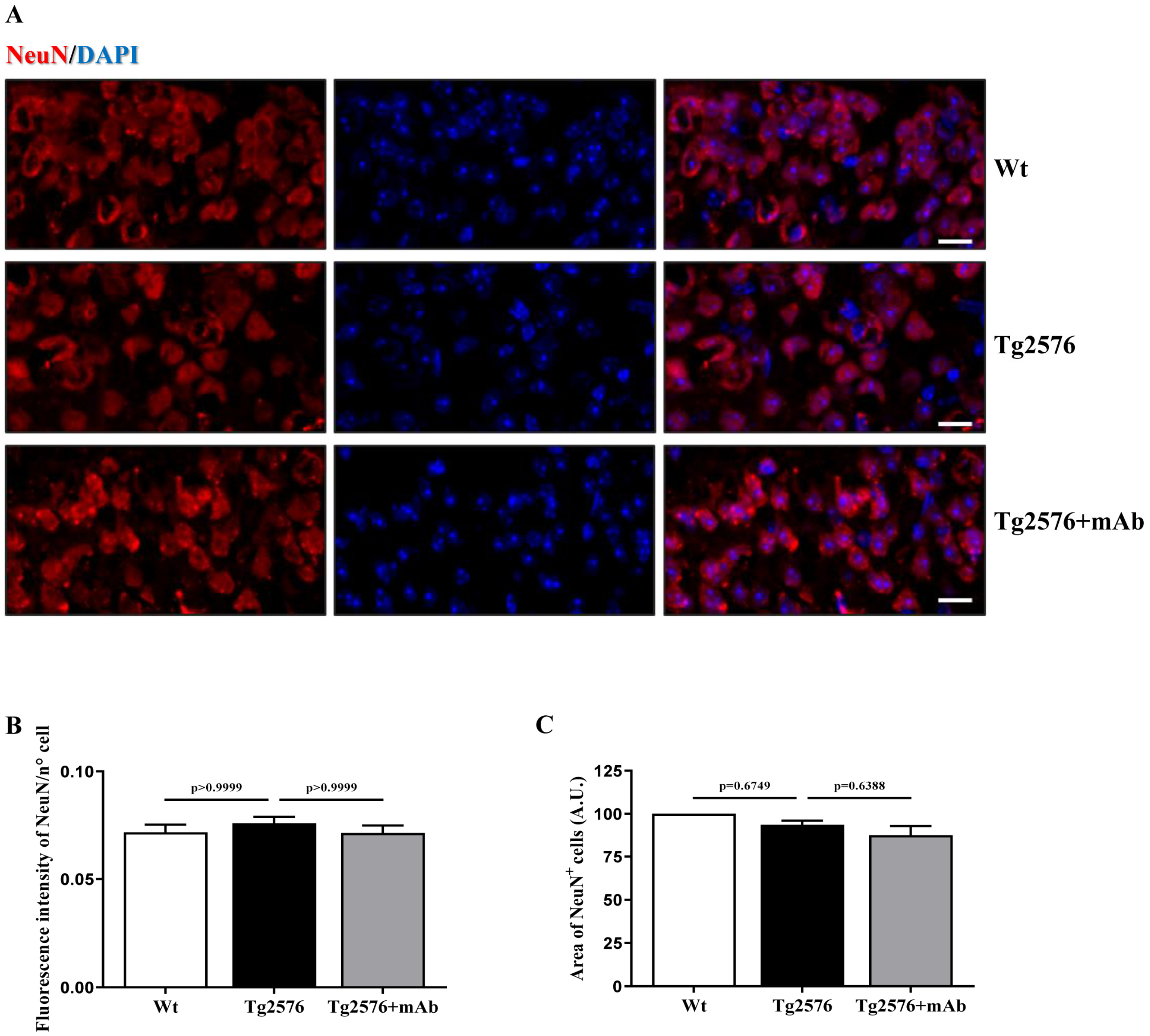

Figure S3. No overt neuron loss and gross histopathological alterations are observed in primary visual cortex from 6-month-old Tg2576 AD mice. (**A**) In the left panel: Representative images of immunofluorescence analysis (20X) showing the diffuse labeling (arrow) of NeuN (red channel) in primary visual cortex (V1) from animals (n = 4 animals per each group, 2 males and 2 females for each experimental condition) of three experimental groups (littermate wild-type, naive/vehicle-treated Tg2576, Tg2576+mAb). In the middle panel: Nuclei were counterstained with DAPI (blue channel). In the right panel: Two-color overlay is shown. NeuN is preferentially localized in the cell nucleoplasm, but a sizeable amount of protein in post-mitotic neurons is also distributed to the proximal cytoplasm [107]. Scale bar = 25 μm. (**B**) Fluorescence intensity quantification of the NeuN staining in the V1 area from three experimental groups (sample size: analyzed neurons/animal = 1085, n = 4). Values are from at least three independent experiments and statistically significant differences were calculated by one-way ANOVA followed by Bonferroni's post-hoc test for multiple comparisons among more than two groups. $p < 0.05$ was accepted as statistically significant. (**C**) Image J measurement of area of NeuN$^+$ cells show no significant variation among three experimental groups (sample size: analyzed neurons/animal = 490, n = 4). Values are from at least three independent experiments and statistically significant differences were calculated by one-way ANOVA followed by Bonferroni's post hoc test for multiple comparisons among more than two groups. $p < 0.05$ was accepted as statistically significant. A.U. = arbitrary units.
